# Supplementary material for: Leveraging Configuration Interaction Singles for Qualitative Descriptions of Ground and Excited States: State-Averaging, Linear-Response, and Spin-Projection
Source: J Chem Theory Comput. 2026 Mar 30;22(7):3467–83. doi: 10.1021/acs.jctc.6c00182 (PMC13085252; doi:10.1021/acs.jctc.6c00182)
Supplement: Supplementary file 1 [file ct6c00182_si_001.pdf]

Supporting Information for "Leveraging configuration interaction singles for qualitative descriptions of ground and excited states: state-averaging, linear-response, and spin-projection"

Takashi Tsuchimochi*
College of Engineering, Shibaura Institute of Technology, 3-7-5 Toyosu, Koto-ku, Tokyo 135-8548 Japan and Institute for Molecular Science, 38 Nishigonaka, Myodaiji, Okazaki 444-8585 Japan

Benjamin Mokhtar
Graduate School of Engineering and Science, Shibaura Institute of Technology, 3-7-5 Toyosu, Koto-ku, Tokyo 135-8548 Japan

Contents

S1. Review of SUHF and ECIS S2
A. SUHF S2
1. Projection operator S2
2. SUHF and related intermediates S2
B. ECIS S3
1. Energy and overlap S3
2. Spin-incomplete 1PDM and 2PDM S4

S2. State-Averaged Parametrization of ECIS States S4

S3. SAECIS Gradients S6
A. CI gradients ${}^{\rm c}g_\mu^I$ S6
B. Orbital gradient couplings ${}^{\rm o}g_{ai}^{I^*K}$ S6

S4. SAECIS Hessian S7
A. Contraction of ${}^{\rm oo}H_{ai,bj}$ with ${}^{\rm o}x_{bj}$ S8
B. Contraction of ${}^{\rm oc}H_{ai,\mu}^I$ with ${}^{\rm c}x_\mu^I$ and ${}^{\rm o}x_{ai}$ S10
C. Contraction of ${}^{\rm cc}H_{\mu\nu}^{IJ}$ with ${}^{\rm c}x_\nu^I$ S12

S5. Double CIS S12
A. Working equation for spin-projected DCIS (EDCIS) S12

S6. Simplified expressions for non-projected SA-CIS and DCIS S13
A. Energy S13
B. Orbital and CI gradients S14
1. ${}^{\rm o}g_{ai}$ and ${}^{\rm o}g_{ai}^{IK}$ S14
2. ${}^{\rm c}g_\mu$ S14
C. Hessian contraction S15
1. ${}^{\rm cc}H_{\mu,\nu}y_\nu$ S15
2. ${}^{\rm oc}H_{ai,\nu}y_\nu$ S16
3. ${}^{\rm co}H_{\mu,bj}x_{bj}$ S16
4. ${}^{\rm oo}H_{ai,bj}x_{bj}$ S17

*Electronic address: tsuchimochi@gmail.com

S1. Review of SUHF and ECIS

A. SUHF

We first recapitulate the SUHF energy and its orbital gradient. Here, all matrices have the dimension of full spin orbitals, unless otherwise noted.

1. *Projection operator*

A projection operator $\hat{P}$ may be generally written as

$$\hat{P} = \int d\Omega w(\Omega)\hat{R}(\Omega) \tag{S1}$$

For spin-projection, $\hat{R}(\Omega)$ corresponds to spin-rotation operator and $\Omega = (\alpha, \beta, \gamma)$ is the Euler angle,

$$\hat{R}(\Omega) = e^{-i\alpha\hat{S}_z} e^{-i\beta\hat{S}_y} e^{-i\gamma\hat{S}_z} \tag{S2}$$

The integral in Eq.(S1) can be approximated by numerical integration with N_{grid} grid points,

$$\hat{P} \approx \sum_{g}^{N_{\text{grid}}} w_g \hat{R}_g \tag{S3}$$

In SUHF and ECIS, the underlying broken-symmetry states (UHF and UCIS) are an eigenfunction of $\hat{S}_z$. Hence, upon the application of $\hat{P}$ or $\hat{R}(\Omega)$, the rotations around the z-axis, $e^{-i\alpha\hat{S}_z}$ and $e^{-i\gamma\hat{S}_z}$ can be replaced by a constant, and can be effectively removed after appropriate normalization. Therefore, in the following, we will use

$$\hat{R}_g \equiv e^{-i\hat{S}_y\beta_g} \tag{S4}$$

in place of $\hat{R}_g$ in Eq.(S3). In the atomic orbital (AO) basis, $\{|\chi_1^\alpha\rangle, \cdots, |\chi_M^\alpha\rangle, |\chi_1^\beta\rangle, \cdots, |\chi_M^\beta\rangle\}$, the matrix representation of $\hat{R}_g$ is

$$\mathbf{R}_{g,\text{AO}} = \begin{pmatrix} \cos\left(\frac{\beta_g}{2}\right)\mathbf{S}^{\text{AO}} & -\sin\left(\frac{\beta_g}{2}\right)\mathbf{S}^{\text{AO}} \\ \sin\left(\frac{\beta_g}{2}\right)\mathbf{S}^{\text{AO}} & \cos\left(\frac{\beta_g}{2}\right)\mathbf{S}^{\text{AO}} \end{pmatrix} \tag{S5}$$

where $S_{\mu\nu}^{\text{AO}} = \langle\chi_\mu|\chi_\nu\rangle$ is the $M \times M$ overlap matrix of the atomic orbitals.

2. *SUHF and related intermediates*

The SUHF energy is given by

$$E_{\text{SUHF}} = \frac{\langle\Phi_0|\hat{P}^\dagger\hat{H}\hat{P}|\Phi_0\rangle}{\langle\Phi_0|\hat{P}^\dagger\hat{P}|\Phi_0\rangle} = \frac{\langle\Phi_0|\hat{H}\hat{P}|\Phi_0\rangle}{\langle\Phi_0|\hat{P}|\Phi_0\rangle} \tag{S6}$$

To evaluate this equation, we first show how the squared norm (denominator) can be calculated.

The matrix representation of spin-rotation operator at the grid angle β_g in the molecular-orbital (MO) basis $|\phi_p\rangle$ is

$$\begin{aligned}(\mathbf{R}_g)_{pq} &= \langle\phi_p|\hat{R}_g|\phi_q\rangle \\ &= \left(\mathbf{C}^\dagger\mathbf{R}_{g,\text{AO}}\mathbf{C}\right)_{pq}\end{aligned} \tag{S7}$$

For convenience, we align the full-spin MO coefficient matrix $\mathbf{C}$ as

$$\mathbf{C} = \begin{pmatrix} \mathbf{C}^{\alpha,\text{o}} & \mathbf{0} & \mathbf{C}^{\alpha,\text{v}} & \mathbf{0} \\ \mathbf{0} & \mathbf{C}^{\beta,\text{o}} & \mathbf{0} & \mathbf{C}^{\beta,\text{v}} \end{pmatrix} \tag{S8}$$

where α and β are usual electron spins and "o" and "v" indicate the occupied and virtual orbitals of $|\Phi_0\rangle$. Then, $\mathbf{R}_g$ has the following blocks;

$$\mathbf{R}_g = \begin{pmatrix} \mathbf{R}_g^{\text{oo}} & \mathbf{R}_g^{\text{ov}} \\ \mathbf{R}_g^{\text{vo}} & \mathbf{R}_g^{\text{vv}} \end{pmatrix} \tag{S9}$$

It is worthwhile to mention that each block has sub-blocks separated by spins according to the structure of $\mathbf{C}$ in Eq.(S8). The overlap between the determinant $|\Phi_0\rangle$ and rotated determinant $\hat{R}_g|\Phi_0\rangle$ is given by

$$\langle\Phi_0|\hat{R}_g|\Phi_0\rangle = \det(\mathbf{R}_g^{\rm oo}) \tag{S10}$$

With Eq.(S10), one can now evaluate the SUHF norm noting Eq.(S3):

$$\langle\Phi_0|\hat{P}|\Phi_0\rangle = \sum_g w_g \langle\Phi_0|\hat{R}_g|\Phi_0\rangle \tag{S11}$$

This leads to the SUHF energy expression as follows:

$$E_{\rm SUHF} = \frac{\langle\Phi_0|\hat{H}\hat{P}|\Phi_0\rangle}{\langle\Phi_0|\hat{P}|\Phi_0\rangle} = \sum_g w_g \frac{\langle\Phi_0|\hat{R}_g|\Phi_0\rangle}{\langle\Phi_0|\hat{P}|\Phi_0\rangle}\frac{\langle\Phi_0|\hat{H}\hat{R}_g|\Phi_0\rangle}{\langle\Phi_0|\hat{R}_g|\Phi_0\rangle} = \sum_g w_g n_g \epsilon_g \tag{S12}$$

where we have defined

$$n_g = \frac{\langle\Phi_0|\hat{R}_g|\Phi_0\rangle}{\langle\Phi_0|\hat{P}|\Phi_0\rangle} \tag{S13}$$

$$\epsilon_g = \frac{\langle\Phi_0|\hat{H}\hat{R}_g|\Phi_0\rangle}{\langle\Phi_0|\hat{R}_g|\Phi_0\rangle} = \frac{1}{2}{\rm Tr}\left[\mathbf{h}\boldsymbol{\rho}_g + \mathbf{F}_g\boldsymbol{\rho}_g\right] \tag{S14}$$

$$(\boldsymbol{\rho}_g)_{pq} = \frac{\langle\Phi_0|a_q^\dagger a_p\hat{R}_g|\Phi_0\rangle}{\langle\Phi_0|\hat{R}_g|\Phi_0\rangle} \tag{S15}$$

$$(\boldsymbol{\eta}_g)_{pq} = \delta_{pq} - (\boldsymbol{\rho}_g)_{pq} \tag{S16}$$

$$\mathbf{F}_g = \mathbf{h} + \mathbf{G}[\boldsymbol{\rho}_g] \tag{S17}$$

$$\tag{S18}$$

Here, h_{pq} are the one-electron integrals and

$$(\mathbf{G}[\mathbf{M}])_{pq} \equiv \sum_{rs}\langle pr||qs\rangle M_{sr} \tag{S19}$$

It is convenient to define the following intermediate matrices

$$\boldsymbol{\mathcal{W}}_g = \begin{pmatrix} (\mathbf{R}_g^{\rm oo})^{-1} & (\mathbf{R}_g^{\rm oo})^{-1}\mathbf{R}_g^{\rm ov} \\ \mathbf{R}_g^{\rm vo}(\mathbf{R}_g^{\rm oo})^{-1} & \mathbf{R}_g^{\rm vv} - \mathbf{R}_g^{\rm vo}(\mathbf{R}_g^{\rm oo})^{-1}\mathbf{R}_g^{\rm ov} \end{pmatrix} \tag{S20}$$

$$\boldsymbol{\mathcal{L}}_g = \begin{pmatrix} \boldsymbol{\mathcal{W}}_g^{\rm oo} & \mathbf{0}^{\rm ov} \\ -\boldsymbol{\mathcal{W}}_g^{\rm vo} & \mathbf{I}^{\rm vv} \end{pmatrix} \tag{S21}$$

$$\boldsymbol{\mathcal{R}}_g = \begin{pmatrix} \mathbf{I}^{\rm oo} & \mathbf{0}^{\rm ov} \\ \boldsymbol{\mathcal{W}}_g^{\rm vo} & \boldsymbol{\mathcal{W}}_g^{\rm vv} \end{pmatrix} \tag{S22}$$

Using these results,

$$\boldsymbol{\rho}_g = \begin{pmatrix} \mathbf{I}^{\rm oo} & \mathbf{0}^{\rm ov} \\ \boldsymbol{\mathcal{W}}_g^{\rm vo} & \mathbf{0}^{\rm vv} \end{pmatrix} \tag{S23}$$

The SUHF energy is variationally minimized by enforcing the virtual-occupied block of the following Fock matrix to be zero:

$$\mathbf{F}^{\rm SUHF} = \sum_g w_g n_g \Big[(\epsilon_g - E_{\rm SUHF})\boldsymbol{\mathcal{W}}_g + \boldsymbol{\mathcal{L}}_g\mathbf{F}_g\boldsymbol{\mathcal{R}}_g\Big] \tag{S24}$$

B. ECIS

1. *Energy and overlap*

Let us define the following matrix elements

$$H_{IK} = \langle 0_I|\hat{H}\hat{P}|0_K\rangle = \sum_g w_g n_g H_g^{IK} \tag{S25}$$

$$N_{IK} = \langle 0_I|\hat{P}|0_K\rangle = \sum_g w_g n_g N_g^{IK} \tag{S26}$$

where $|0_I\rangle$ is the underlying broken-symmetry expansion, which is given by the standard unrestricted CIS wave function,

$$|0_I\rangle = \sum_\mu c_\mu^I |\Phi_\mu\rangle \tag{S27}$$

The ECIS energy of the Ith state is given by

$$E_{0,I} = \frac{H_{II}}{N_{II}} \tag{S28}$$

At each grid g, the couplings H_g^{IK} and N_g^{IK} are evaluated as

$$H_g^{IK} = \frac{\langle 0_I|\hat{H}\hat{R}_g|0_K\rangle}{\langle\Phi_0|\hat{R}_g|\Phi_0\rangle} = \epsilon_g N_g^{IK} + \mathrm{Tr}\left[\mathbf{F}_g\boldsymbol{\gamma}_g^{IK} + \mathbf{G}[\boldsymbol{\mathcal{D}}_g^I]\boldsymbol{\mathcal{C}}_g^K\right] \tag{S29}$$

$$N_g^{IK} = \frac{\langle 0_I|\hat{R}_g|0_K\rangle}{\langle\Phi_0|\hat{R}_g|\Phi_0\rangle} = \tilde{d}_0^I\tilde{c}_0^K + \mathrm{Tr}[\boldsymbol{\mathcal{D}}_g^I\boldsymbol{\mathcal{C}}_g^K] \tag{S30}$$

using the following intermediates

$$\tilde{c}_0^I = c_0^I + (\boldsymbol{\mathcal{W}}_g)_{kc}c_{ck}^I \tag{S31}$$

$$\tilde{d}_0^I = c_0^I + (\boldsymbol{\mathcal{W}}_g)_{ck}{}^{\mathrm{c}}c_{ck}^I \tag{S32}$$

$$(\boldsymbol{\mathcal{C}}_g^I)_{pq} = \sum_{ai}(\boldsymbol{\mathcal{R}}_g)_{pa}c_{ai}^I(\boldsymbol{\mathcal{L}}_g)_{iq} \tag{S33}$$

$$(\boldsymbol{\mathcal{D}}_g^I)_{pq} = \sum_{ai}(\boldsymbol{\mathcal{R}}_g)_{pi}c_{ai}^I(\boldsymbol{\mathcal{L}}_g)_{aq} \tag{S34}$$

$$\begin{aligned}(\boldsymbol{\gamma}_g^{IK})_{pq} &= \frac{\langle 0_I|\{a_q^\dagger a_p\}_g\hat{R}_g|0_K\rangle}{\langle\Phi_0|\hat{R}_g|\Phi_0\rangle} \\ &= \left(\tilde{d}_0^I\boldsymbol{\mathcal{C}}_g^K + \tilde{c}_0^K\boldsymbol{\mathcal{D}}_g^I - \boldsymbol{\mathcal{D}}_g^I\boldsymbol{\mathcal{C}}_g^K + \boldsymbol{\mathcal{C}}_g^K\boldsymbol{\mathcal{D}}_g^I\right)_{pq}\end{aligned} \tag{S35}$$

2. Spin-incomplete 1PDM and 2PDM

The spin-incomplete 1PDM and 2PDM of the I th ECIS state are expressed as

$$P_{qp}^I := \langle 0_I|a_p^\dagger a_q\hat{P}|0_I\rangle = \sum_g w_g n_g(\mathbf{P}_g^I)_{qp} \tag{S36}$$

$$P_{qp,sr}^I = \langle 0_I|a_p^\dagger a_q^\dagger a_s a_r\hat{P}|0_I\rangle = \sum_g w_g n_g(\mathbf{P}_g^I)_{qp,sr} \tag{S37}$$

where

$$(\mathbf{P}_g^I)_{pq} = N_g^{II}(\boldsymbol{\rho}_g)_{pq} + (\boldsymbol{\gamma}_g^{II})_{pq} \tag{S38}$$

$$(\mathbf{P}_g^I)_{pq,rs} = \mathcal{P}(pq)\mathcal{P}(rs)\left(\frac{1}{2}N_g^{II}(\boldsymbol{\rho}_g)_{rp}(\boldsymbol{\rho}_g)_{sq} + (\boldsymbol{\gamma}_g^{II})_{rp}(\boldsymbol{\rho}_g)_{sq} + (\boldsymbol{\mathcal{C}}_g^I)_{rp}(\boldsymbol{\mathcal{D}}_g^I)_{sq}\right) \tag{S39}$$

where $\mathcal{P}(pq)$ is the anti-symmetrizer. Obviously, the ECIS energy is given by

$$E_{0,I} = \sum_{pq}h_{pq}P_{qp}^I + \frac{1}{4}\sum_{pqrs}\langle pq||rs\rangle P_{qp,sr}^I \tag{S40}$$

However, these PDMs are spin-incomplete because only the ket state is projected. While this fact does not affect the final result in most cases (except when spin-dependent property is required such as spin density), one can use the Wigner-Eckart theorem to obtain the spin-projected density matrices, if needed.

S2. State-Averaged Parametrization of ECIS States

We parametrize each Ith ECIS state $|\tilde{0}_I\rangle$ in terms of (i) the anti-Hermitian orbital-rotation generator

$${}^{\mathrm{o}}\hat{\lambda} = \sum_{ai}{}^{\mathrm{o}}\lambda_{ai}\,\hat{E}_{ai}^-, \tag{S41}$$

and (ii) a CI perturbation $|{}^{c}\boldsymbol{\lambda}^{I}\rangle$, which displaces the reference CI coefficients $\mathbf{c}^{I}$ within the CI space as

$$|{}^{c}\boldsymbol{\lambda}^{I}\rangle = \sum_{\mu} {}^{c}\lambda_{\mu}^{I}\,|\Phi_{\mu}\rangle. \tag{S42}$$

Here ${}^{o}\boldsymbol{\lambda} = \{{}^{o}\lambda_{ai}\}$ are orbital-rotation parameters, and ${}^{c}\boldsymbol{\lambda}^{I} = \{{}^{c}\lambda_{\mu}^{I}\}$ describe linear response of the CI expansion within a fixed determinant basis $\{|\Phi_{\mu}\rangle\}$.

It is advantageous to keep the CI perturbation within the complement of the reference subspace, especially after an orbital rotation. However, because the spin-projection operator $\hat{P}$ does not commute with (spin-unrestricted) orbital rotations,

$$e^{{}^{o}\hat{\lambda}}\,\hat{P}\,e^{-{}^{o}\hat{\lambda}} \neq \hat{P}, \tag{S43}$$

the simple ansatz $|\tilde{0}_{I}[{}^{o}\boldsymbol{\lambda}, {}^{c}\boldsymbol{\lambda}^{I}]\rangle$ cannot in general remain orthonormal for arbitrary ${}^{o}\boldsymbol{\lambda}$ and ${}^{c}\boldsymbol{\lambda}^{I}$.

To treat this difficulty we construct a projector onto the complement CI space that is exactly orthogonal to the orbitally rotated and projected references $\{\hat{P}e^{-{}^{o}\hat{\lambda}}|0_{I}\rangle\}_{I=1}^{n}$. Let

$$\mathcal{N}_{IK}({}^{o}\boldsymbol{\lambda}) = \langle 0_{I}|e^{+{}^{o}\hat{\lambda}}\hat{P}e^{-{}^{o}\hat{\lambda}}|0_{K}\rangle \tag{S44}$$

be their overlap. We then define the Hermitian, idempotent projector

$$\hat{\mathcal{Q}}[{}^{o}\boldsymbol{\lambda}] = 1 - \sum_{I,K} \hat{P}e^{-{}^{o}\hat{\lambda}}|0_{I}\rangle(\boldsymbol{\mathcal{N}}({}^{o}\boldsymbol{\lambda})^{-1})_{IK}\langle 0_{K}|e^{+{}^{o}\hat{\lambda}}\hat{P} \tag{S45}$$

onto the subspace orthogonal to the entire set $\{\hat{P}e^{-{}^{o}\hat{\lambda}}|0_{I}\rangle\}_{I=1}^{n}$ for arbitrary ${}^{o}\boldsymbol{\lambda}$.

Using this operator, we define the variational ansatz

$$|\tilde{0}_{I}[{}^{o}\boldsymbol{\lambda}, {}^{c}\boldsymbol{\lambda}^{I}]\rangle = \hat{P}e^{-{}^{o}\hat{\lambda}}\Big[|0_{I}\rangle + e^{{}^{o}\hat{\lambda}}\hat{\mathcal{Q}}[{}^{o}\boldsymbol{\lambda}]e^{-{}^{o}\hat{\lambda}}|{}^{c}\boldsymbol{\lambda}^{I}\rangle\Big]. \tag{S46}$$

The unitary transformation $e^{{}^{o}\hat{\lambda}}\hat{\mathcal{Q}}e^{-{}^{o}\hat{\lambda}}$ ensures that the CI perturbation is orthogonal to the orbitally rotated, projected references, while allowing the orbital rotation $e^{-{}^{o}\hat{\lambda}}$ to act first on every component.

Although the projected states $\{|\tilde{0}_{I}\rangle\}$ are not mutually orthonormal, this is not a limitation: their mutual non-orthogonality is exactly accounted for by the generalized overlap matrix

$$\mathcal{N}_{IJ}[{}^{o}\boldsymbol{\lambda}, {}^{c}\boldsymbol{\lambda}^{I}, {}^{c}\boldsymbol{\lambda}^{J}] = \langle \tilde{0}_{I}[{}^{o}\boldsymbol{\lambda}, {}^{c}\boldsymbol{\lambda}^{I}]|\tilde{0}_{J}[{}^{o}\boldsymbol{\lambda}, {}^{c}\boldsymbol{\lambda}^{J}]\rangle \tag{S47}$$

and the corresponding Hamiltonian matrix

$$\mathcal{H}_{IJ}[{}^{o}\boldsymbol{\lambda}, {}^{c}\boldsymbol{\lambda}^{I}, {}^{c}\boldsymbol{\lambda}^{J}] = \langle \tilde{0}_{I}[{}^{o}\boldsymbol{\lambda}, {}^{c}\boldsymbol{\lambda}^{I}]|\hat{H}|\tilde{0}_{J}[{}^{o}\boldsymbol{\lambda}, {}^{c}\boldsymbol{\lambda}^{J}]\rangle \tag{S48}$$

which include all cross terms involving $\hat{\mathcal{Q}}$. The true orthonormal states and energies are obtained by solving the generalized eigenvalue problem

$$\boldsymbol{\mathcal{H}}\mathbf{V} = \boldsymbol{\mathcal{N}}\mathbf{V}\mathbf{E}. \tag{S49}$$

Because we are interested in the state-averaged energy $\frac{1}{n}\,\mathrm{Tr}\,\mathbf{E}$, we can work entirely within the non-orthogonal basis and write

$$E_{\mathrm{ave}} = \frac{1}{n}\,\mathrm{Tr}\left[\boldsymbol{\mathcal{H}}\boldsymbol{\mathcal{N}}^{-1}\right], \tag{S50}$$

which is variationally equivalent and numerically convenient.

Having defined the averaged energy and required matrix components, we take the first and second derivatives of E_{ave} with respect to both ${}^{o}\boldsymbol{\lambda}$ and $\{{}^{c}\boldsymbol{\lambda}_{I}\}$ at the unrotated/unperturbed reference $\{|\tilde{0}_{I}[\mathbf{0}, \mathbf{0}]\rangle\} \equiv \{|0_{I}\rangle\}$. To do so, we first consider the general result:

$$\begin{aligned}\left.\frac{\partial E_{\mathrm{ave}}}{\partial\theta}\right|_{\theta=0} &= \frac{1}{n}\sum_{I}\left.\frac{\partial(\boldsymbol{\mathcal{H}}\boldsymbol{\mathcal{N}}^{-1})_{II}}{\partial\theta}\right|_{\theta=0} = \sum_{IKL}\left(\frac{\partial\mathcal{H}_{IL}}{\partial\theta}\delta_{KL} - (\boldsymbol{\mathcal{H}}\boldsymbol{\mathcal{N}}^{-1})_{IK}\frac{\partial\mathcal{N}_{KL}}{\partial\theta}\right)\mathcal{N}_{LI}^{-1}\Bigg|_{\theta=0}\\ &= \sum_{I}\left(\sum_{K}\frac{\partial\mathcal{H}_{IK}}{\partial\theta}\mathcal{N}_{KI}^{-1} - \sum_{KL}(\boldsymbol{\mathcal{H}}\boldsymbol{\mathcal{N}}^{-1})_{IK}\frac{\partial\mathcal{N}_{KL}}{\partial\theta}\mathcal{N}_{LI}^{-1}\right)\Bigg|_{\theta=0}\end{aligned} \tag{S51}$$

and

$$\left.\frac{\partial^{2}E_{\mathrm{ave}}}{\partial\theta\partial\theta'}\right|_{\theta=0,\theta'=0} = \frac{1}{n}\sum_{IL}\Big(\frac{\partial^{2}\mathcal{H}_{IL}}{\partial\theta\partial\theta'} - (\boldsymbol{\mathcal{H}}\boldsymbol{\mathcal{N}}^{-1})_{IJ}\frac{\partial^{2}\mathcal{N}_{JL}}{\partial\theta\partial\theta'} - \frac{\partial(\boldsymbol{\mathcal{H}}\boldsymbol{\mathcal{N}}^{-1})_{IK}}{\partial\theta}\frac{\partial\mathcal{N}_{KL}}{\partial\theta'} - \frac{\partial(\boldsymbol{\mathcal{H}}\boldsymbol{\mathcal{N}}^{-1})_{IK}}{\partial\theta'}\frac{\partial\mathcal{N}_{KL}}{\partial\theta}\Big)\mathcal{N}_{LI}^{-1}\Bigg|_{\theta=0,\theta'=0} \tag{S52}$$

By conveniently choosing $\langle 0_I|\hat{P}|0_J\rangle = \delta_{IJ}$ and $\langle 0_I|\hat{H}\hat{P}|0_J\rangle = E_{0,I}\delta_{IJ}$, we obtain

$$\mathcal{H}_{IJ}[\mathbf{0},\mathbf{0},\mathbf{0}] = \langle 0_I|\hat{H}\hat{P}|0_I\rangle = E_{0,I}\delta_{IJ} \tag{S53}$$

$$\mathcal{N}_{IJ}[\mathbf{0},\mathbf{0},\mathbf{0}] = \delta_{IJ} \tag{S54}$$

where $E_{0,I}$ is the energy expectation value of the current $|0_I\rangle$. In this basis, these can be simplified as

$$\left.\frac{\partial E_{\text{ave}}}{\partial\theta}\right|_{\theta=0} = \sum_I \Big(\frac{\partial\mathcal{H}_{II}}{\partial\theta} - E_{0,I}\frac{\partial\mathcal{N}_{II}}{\partial\theta}\Big) \tag{S55}$$

and

$$\left.\frac{\partial^2 E_{\text{ave}}}{\partial\theta\partial\theta'}\right|_{\theta=0,\theta'=0} = \sum_I \Big(\frac{\partial^2\mathcal{H}_{II}}{\partial\theta\partial\theta'} - E_{0,I}\frac{\partial^2\mathcal{N}_{II}}{\partial\theta\partial\theta'} - \sum_K\big(\frac{\partial\mathcal{H}_{IK}}{\partial\theta} - E_{0,I}\frac{\partial\mathcal{N}_{IK}}{\partial\theta}\big)\frac{\partial\mathcal{N}_{KI}}{\partial\theta'} - \sum_K\big(\frac{\partial\mathcal{H}_{IK}}{\partial\theta'} - E_{0,I}\frac{\partial\mathcal{N}_{IK}}{\partial\theta'}\big)\frac{\partial\mathcal{N}_{KI}}{\partial\theta}\Big) \tag{S56}$$

Noting that $\hat{\mathcal{Q}}$ possesses the dependence on ${}^{\text{o}}\boldsymbol{\lambda}$ but not on ${}^{\text{c}}\boldsymbol{\lambda}^I$,

$$\left.\frac{\partial\hat{\mathcal{Q}}[{}^{\text{o}}\boldsymbol{\lambda}]}{\partial{}^{\text{o}}\lambda_{ai}}\right|_{\boldsymbol{\lambda}=\mathbf{0}} = \sum_J \Big(\hat{E}_{ai}^-|0_J\rangle\langle 0_J|\hat{P} - \hat{P}|0_J\rangle\langle 0_J|\hat{E}_{ai}^-\Big) + \sum_{JK}\hat{P}|0_J\rangle\langle 0_J|(\hat{E}_{ai}^-\hat{P} - \hat{P}\hat{E}_{ai}^-)|0_K\rangle\langle 0_K|\hat{P} \tag{S57}$$

$$\left.\frac{\partial\hat{\mathcal{Q}}[{}^{\text{o}}\boldsymbol{\lambda}]}{\partial{}^{\text{c}}\lambda_\mu^I}\right|_{\boldsymbol{\lambda}=\mathbf{0}} = 0 \tag{S58}$$

Using these relations, we arrive at the electronic gradient and Hessian of state-averaged ECIS as described in the main text. Below, we further provide detailed working equations.

S3. SAECIS Gradients

A. CI gradients ${}^{\text{c}}g_\mu^I$

The ECIS energy is variationally minimized by optimizing the CI coefficients. This results in the following gradient:

$${}^{\text{c}}g_\mu^I = \frac{1}{n}\Big(\langle\Phi_\mu|(\hat{H}-E_{0,I})\hat{P}|0_I\rangle + \langle 0_I|(\hat{H}-E_{0,I})\hat{P}|\Phi_\mu\rangle\Big) \tag{S59}$$

where we have adopted the definition from the main text ($n = 1$ for the state-specific ECIS) and

$$\langle\Phi_0|(\hat{H}-E_{0,I})\hat{P}|0_I\rangle = \sum_g w_g n_g\Big[(\epsilon_g - E_{0,I})\tilde{c}_0^I + \text{Tr}[\mathbf{F}_g\boldsymbol{\mathcal{C}}_g^I]\Big] \tag{S60}$$

$$\langle\Phi_i^a|(\hat{H}-E_{0,I})\hat{P}|0_I\rangle = \sum_g w_g n_g\Big[(\epsilon_g - E_{0,I})(\tilde{c}_0^I\boldsymbol{\mathcal{W}}_g + \boldsymbol{\mathcal{C}}_g^I) + \text{Tr}[\mathbf{F}_g\boldsymbol{\mathcal{C}}_g^I]\boldsymbol{\mathcal{W}}_g + \tilde{c}_0^I\boldsymbol{\mathcal{L}}_g\mathbf{F}_g\boldsymbol{\mathcal{R}}_g + \boldsymbol{\mathcal{L}}_g\mathbf{F}_g\boldsymbol{\mathcal{C}}_g^I - \boldsymbol{\mathcal{C}}_g^I\mathbf{F}_g\boldsymbol{\mathcal{R}}_g + \boldsymbol{\mathcal{L}}_g\mathbf{G}[\boldsymbol{\mathcal{C}}_g^I]\boldsymbol{\mathcal{R}}_g\Big]_{ai} \tag{S61}$$

and

$$\langle 0_I|(\hat{H}-E_{0,I})\hat{P}|\Phi_0\rangle = \sum_g w_g n_g\Big[(\epsilon_g - E_{0,I})\tilde{d}_0^I + \text{Tr}[\mathbf{F}_g\boldsymbol{\mathcal{D}}_g^I]\Big] \tag{S62}$$

$$\langle 0_I|(\hat{H}-E_{0,I})\hat{P}|\Phi_i^a\rangle = \sum_g w_g n_g\Big[(\epsilon_g - E_{0,I})(\tilde{d}_0\boldsymbol{\mathcal{W}}_g + \boldsymbol{\mathcal{D}}_g^I) + \text{Tr}[\mathbf{F}_g\boldsymbol{\mathcal{D}}_g^I]\boldsymbol{\mathcal{W}}_g + \tilde{d}_0^I\boldsymbol{\mathcal{L}}_g\mathbf{F}_g\boldsymbol{\mathcal{R}}_g + \boldsymbol{\mathcal{D}}_g^I\mathbf{F}_g\boldsymbol{\mathcal{R}}_g - \boldsymbol{\mathcal{L}}_g\mathbf{F}_g\boldsymbol{\mathcal{D}}_g^I + (\boldsymbol{\mathcal{L}}_g\mathbf{G}[\boldsymbol{\mathcal{D}}_g^I]\boldsymbol{\mathcal{R}}_g)\Big]_{ia} \tag{S63}$$

Eqs.(S62) and (S63) are the complex conjugates of Eqs.(S60) and (S61) and therefore do not have to be explicitly computed. However, they yield different contributions to the relaxed density matrices when the derivatives are taken.

B. Orbital gradient couplings ${}^{\text{o}}g_{ai}^{I^*K}$

As shown in the main text, we have defined

$${}^{\text{o}}g_{ai}^{I^*K} = \langle 0_I|\Big[\hat{E}_{ai}^-, (\hat{H}-E_{0,I})\hat{P}\Big]|0_K\rangle \tag{S64}$$

where superscript * on I indicates that the energy of the Ith state is used to build the matrix element. We note that, for the first derivative, i.e., the orbital gradient of the energy and/or averaged energy, only the diagonals ${}^{\rm o}g_{ai}^{I^*I}$ are relevant. Namely, for the state-specific ECIS, the orbital gradient is ${}^{\rm o}g_{ai}^{I^*I}$, and for the state-averaged ECIS, it is $\frac{1}{n}\sum_I {}^{\rm o}g_{ai}^{I^*I}$. The coupling between I and K will become later important for the second derivative, and therefore we will derive its working equation here.

To evaluate this term, we decompose it to different contributions:

$$ {}^{\rm o}g_{ai}^{I^*K} = L_{ia}^{I^*K} - L_{ai}^{I^*K} - \bar{L}_{ia}^{I^*K} + \bar{L}_{ai}^{I^*K} \tag{S65} $$

with

$$ L_{pq}^{I^*K} := \langle 0_I|\hat{E}_{qp}(\hat{H}-E_{0,I})\hat{P}|0_K\rangle \tag{S66} $$
$$ \bar{L}_{pq}^{I^*K} := \langle 0_I|(\hat{H}-E_{0,I})\hat{P}\hat{E}_{qp}|0_K\rangle = (L_{qp}^{KI^*})^* \tag{S67} $$

$L_{pq}^{I^*K}$ and $\bar{L}_{pq}^{I^*K}$ are written as the following numerical integration

$$ \mathbf{L}^{I^*K} = \sum_g w_g n_g \mathbf{L}_g^{I^*K} \tag{S68} $$
$$ \bar{\mathbf{L}}^{I^*K} = \sum_g w_g n_g \left[\mathbf{R}_g^\dagger \bar{\mathbf{L}}_g^{I^*K}\mathbf{R}_g\right] \tag{S69} $$

with

$$ \begin{aligned} \mathbf{L}_g^{I^*K} =& \boldsymbol{\gamma}_g^{IK}(\epsilon_g - E_{0,I}) + \boldsymbol{\rho}_g(H_g^{IK} - N_g^{IK}E_{0,I}) - \boldsymbol{\gamma}_g^{IK}\mathbf{F}_g\boldsymbol{\rho}_g + \boldsymbol{\eta}_g\mathbf{F}_g\boldsymbol{\gamma}_g^{IK} + \boldsymbol{\eta}_g\mathbf{F}_g\boldsymbol{\rho}_g N_g^{IK} + \boldsymbol{\eta}_g\mathbf{G}[\boldsymbol{\gamma}_g^{IK}]\boldsymbol{\rho}_g \\ &+ \boldsymbol{\mathcal{D}}_g^I\mathrm{Tr}[\mathbf{F}_g\boldsymbol{\mathcal{C}}_g^K] + \boldsymbol{\mathcal{C}}_g^K\mathrm{Tr}[\mathbf{F}_g\boldsymbol{\mathcal{D}}_g^I] - \boldsymbol{\mathcal{D}}_g^I\mathbf{F}_g\boldsymbol{\mathcal{C}}_g^K - \boldsymbol{\mathcal{C}}_g^K\mathbf{F}_g\boldsymbol{\mathcal{D}}_g^I \\ &- \boldsymbol{\mathcal{D}}_g^I\mathbf{G}[\boldsymbol{\mathcal{C}}_g^K]\boldsymbol{\rho}_g - \boldsymbol{\mathcal{C}}_g^K\mathbf{G}[\boldsymbol{\mathcal{D}}_g^I]\boldsymbol{\rho}_g + \boldsymbol{\eta}_g\mathbf{G}[\boldsymbol{\mathcal{C}}_g^K]\boldsymbol{\mathcal{D}}_g^I + \boldsymbol{\eta}_g\mathbf{G}[\boldsymbol{\mathcal{D}}_g^I]\boldsymbol{\mathcal{C}}_g^K \end{aligned} \tag{S70} $$

$$ \begin{aligned} \bar{\mathbf{L}}_g^{I^*K} =& \boldsymbol{\gamma}_g^{IK}(\epsilon_g - E_{0,I}) + \boldsymbol{\rho}_g(H_g^{IK} - E_{0,I}N_g^{IK}) - \boldsymbol{\rho}_g\mathbf{F}_g\boldsymbol{\gamma}_g^{IK} + \boldsymbol{\gamma}_g^{IK}\mathbf{F}_g\boldsymbol{\eta}_g + \boldsymbol{\rho}_g\mathbf{F}_g\boldsymbol{\eta}_g N_g^{IK} + \boldsymbol{\rho}_g\mathbf{G}[\boldsymbol{\gamma}_g^{IK}]\boldsymbol{\eta}_g \\ &+ \boldsymbol{\mathcal{D}}_g^I\mathrm{Tr}[\mathbf{F}_g\boldsymbol{\mathcal{C}}_g^K] + \boldsymbol{\mathcal{C}}_g^K\mathrm{Tr}[\mathbf{F}_g\boldsymbol{\mathcal{D}}_g^I] - \boldsymbol{\mathcal{D}}_g^I\mathbf{F}_g\boldsymbol{\mathcal{C}}_g^K - \boldsymbol{\mathcal{C}}_g^K\mathbf{F}_g\boldsymbol{\mathcal{D}}_g^I \\ &+ \boldsymbol{\mathcal{D}}_g^I\mathbf{G}[\boldsymbol{\mathcal{C}}_g^K]\boldsymbol{\eta}_g + \boldsymbol{\mathcal{C}}_g^K\mathbf{G}[\boldsymbol{\mathcal{D}}_g^I]\boldsymbol{\eta}_g - \boldsymbol{\rho}_g\mathbf{G}[\boldsymbol{\mathcal{C}}_g^K]\boldsymbol{\mathcal{D}}_g^I - \boldsymbol{\rho}_g\mathbf{G}[\boldsymbol{\mathcal{D}}_g^I]\boldsymbol{\mathcal{C}}_g^K \end{aligned} \tag{S71} $$

S4. SAECIS Hessian

The Hessian of SAECIS is derived as

$$ \begin{aligned} {}^{\rm oo}H_{ai,bj} &= \left.\frac{\partial^2 E_{\rm ave}}{\partial\,{}^{\rm o}\lambda_{ai}\partial\,{}^{\rm o}\lambda_{bj}}\right|_{\boldsymbol{\lambda}=\mathbf{0}} \\ &= \frac{1}{n}\sum_I^n {}^{\rm oo}H_{ai,bj}^I \end{aligned} \tag{S72} $$

where we write the orbital Hessian of the Ith ECIS state as

$$ \begin{aligned} {}^{\rm oo}H_{pq,rs}^I =& \frac{1}{2}\langle 0_I|\left[\hat{E}_{pq}^-,\left[\hat{E}_{rs}^-,(\hat{H}-E_{0,I})\hat{P}\right]\right]|0_I\rangle + \frac{1}{2}\langle 0_I|\left[\hat{E}_{rs}^-,\left[\hat{E}_{pq}^-,(\hat{H}-E_{0,I})\hat{P}\right]\right]|0_I\rangle \\ &- \sum_K^n {}^{\rm o}g_{pq}^{IK}\langle 0_K|\left[\hat{E}_{rs}^-,\hat{P}\right]|0_I\rangle - \sum_K^n {}^{\rm o}g_{rs}^{IK}\langle 0_K|\left[\hat{E}_{pq}^-,\hat{P}\right]|0_I\rangle \end{aligned} \tag{S73} $$

For the oc and cc components,

$$ \begin{aligned} {}^{\rm oc}H_{ai,\mu}^I =& \left.\frac{\partial^2 E_{\rm ave}}{\partial\,{}^{\rm o}\lambda_{ai}\partial\,{}^{\rm c}\lambda_\mu^I}\right|_{\boldsymbol{\lambda}=\mathbf{0}} \\ =& \frac{1}{n}\Bigg(\langle 0_I|\left[\hat{E}_{ai}^-,(\hat{H}-E_{0,I})\hat{P}\right]|\Phi_\mu\rangle + \langle\Phi_\mu|\left[\hat{E}_{ai}^-,(\hat{H}-E_{0,I})\hat{P}\right]|0_I\rangle \\ &- \sum_K^n\left({}^{\rm o}g_{ai}^{I^*K}\langle 0_K|\hat{P}|\Phi_\mu\rangle + \langle\Phi_\mu|\hat{P}|0_K\rangle g_{ai}^{KI^*}\right)\Bigg) - {}^{\rm c}g_\mu^I\langle 0_I|\left[\hat{E}_{ai}^-,\hat{P}\right]|0_I\rangle \end{aligned} \tag{S74} $$

$$
\begin{aligned}
{}^{\mathrm{cc}}H^{IJ}_{\mu\nu} &= \left.\frac{\partial^2 E_{\mathrm{ave}}}{\partial\, {}^{\mathrm{c}}\lambda^I_\mu \partial\, {}^{\mathrm{c}}\lambda^J_\nu}\right|_{\boldsymbol{\lambda}=\mathbf{0}} \\
&= \frac{\delta_{IJ}}{n}\Bigg(\langle\Phi_\mu|(\hat{H}-E_{0,I})\hat{P}|\Phi_\nu\rangle - \sum_K^n \langle\Phi_\mu|\hat{P}|0_K\rangle\langle 0_K|(\hat{H}-E_{0,K})\hat{P}|\Phi_\nu\rangle - \sum_K^n \langle\Phi_\mu|(\hat{H}-E_{0,I})\hat{P}|0_K\rangle\langle 0_K|\hat{P}|\Phi_\nu\rangle \\
&+ \langle\Phi_\nu|(\hat{H}-E_{0,I})\hat{P}|\Phi_\mu\rangle - \sum_K^n \langle\Phi_\nu|\hat{P}|0_K\rangle\langle 0_K|(\hat{H}-E_{0,K})\hat{P}|\Phi_\mu\rangle - \sum_K^n \langle\Phi_\nu|(\hat{H}-E_{0,I})\hat{P}|0_K\rangle\langle 0_K|\hat{P}|\Phi_\mu\rangle \Bigg) \\
&= \frac{\delta_{IJ}}{n}\Bigg(\langle\Phi_\mu|(\hat{H}-E_{0,I})\hat{P}|\Phi_\nu\rangle + \langle\Phi_\nu|(\hat{H}-E_{0,I})\hat{P}|\Phi_\mu\rangle \\
&- \sum_K^n \Big(\langle\Phi_\mu|\hat{P}|0_K\rangle\langle 0_K|(\hat{H}-E_{0,K})\hat{P}|\Phi_\nu\rangle + \langle\Phi_\mu|(\hat{H}-E_{0,K})\hat{P}|0_K\rangle\langle 0_K|\hat{P}|\Phi_\nu\rangle \Big) \\
&- \sum_K^n \langle\Phi_\nu|\hat{P}|0_K\rangle\langle 0_K|(\hat{H}-E_{0,K})\hat{P}|\Phi_\mu\rangle - \sum_K^n \Big(\langle\Phi_\nu|(\hat{H}-E_{0,K})\hat{P}|0_K\rangle\langle 0_K|\hat{P}|\Phi_\mu\rangle \Big) \\
&- \sum_K^n (E_{0,K}-E_{0,I})\Big(\langle\Phi_\mu|\hat{P}|0_K\rangle\langle 0_K|\hat{P}|\Phi_\nu\rangle + \langle\Phi_\nu|\hat{P}|0_K\rangle\langle 0_K|\hat{P}|\Phi_\mu\rangle \Big)\Bigg)
\end{aligned} \tag{S75}
$$

In order to facilitate computation, we employ the Davidson algorithm to diagonalize the augmented Hessian or a linear solver for the coupled-perturbed ECIS equation. For the augmented Hessian, a trial vector $\mathbf{x} = \begin{pmatrix} x_0 & {}^{\mathrm{o}}\mathbf{x} & {}^{\mathrm{c}}\mathbf{x}^1 & {}^{\mathrm{c}}\mathbf{x}^2 & \cdots & {}^{\mathrm{c}}\mathbf{x}^n \end{pmatrix}^\top$ is multiplied to ${}^{\mathrm{oo}}\mathbf{H}$, ${}^{\mathrm{oc}}\mathbf{H}$, and ${}^{\mathrm{cc}}\mathbf{H}$

$$ {}^{\mathrm{o}}\sigma_{ai} = {}^{\mathrm{o}}g_{ai}x_0 + \sum_{bj} {}^{\mathrm{oo}}H_{ai,bj}\, {}^{\mathrm{o}}x_{bj} + \sum_{I,\mu} {}^{\mathrm{oc}}H^I_{ai,\mu}\, {}^{\mathrm{c}}x^I_\mu \tag{S76} $$

$$ {}^{\mathrm{c}}\sigma^I_\mu = {}^{\mathrm{c}}g^I_\mu x_0 + \sum_{ai} {}^{\mathrm{co}}H^I_{\mu,ai}\, {}^{\mathrm{o}}x_{ai} + \sum_{\nu} {}^{\mathrm{cc}}H^I_{\mu,\nu}\, {}^{\mathrm{c}}x^I_\nu \tag{S77} $$

and similar contraction is performed for a linear solver algorithm.

Although occupied-occupied and virtual-virtual orbital rotations are redundant, in our derivation, we deliberately use the full spin orbitals p,q,r,s, to make use of anti-symmetry of quantities as well as to keep the simplicity of equations as much as possible in terms of matrix-matrix multiplications. To arrive at the final working equations of (S76) and (S77) where only occupied-virtual rotations are taken into account, one only needs to replace the indices p,q with a,i. We also take advantage of real wave functions of ECIS in the following.

We will not show the matrix elements of the Hessian explicitly, because they can be easily deduced from the sigma-vectors.

A. Contraction of ${}^{\mathrm{oo}}H_{ai,bj}$ with ${}^{\mathrm{o}}x_{bj}$

To evaluate ${}^{\mathrm{oo}}H^I_{pq,rs}$, we define

$$ P^{IK}_{qp} = \langle 0_I|\hat{E}_{pq}\hat{P}|0_K\rangle \tag{S78} $$

$$ \bar{P}^{IK}_{qp} = \langle 0_I|\hat{P}\hat{E}_{pq}|0_K\rangle = P^{KI*}_{pq} \tag{S79} $$

$$ {}^{\mathrm{oo}}A^I_{qp,rs} = \langle 0_I|\hat{E}_{pq}(\hat{H}-E_{0,I})\hat{P}\hat{E}_{rs}|0_I\rangle = \langle 0_I|\hat{E}_{sr}(\hat{H}-E_{0,I})\hat{P}\hat{E}_{qp}|0_I\rangle^* = {}^{\mathrm{oo}}A^{I*}_{rs,qp} \tag{S80} $$

$$ {}^{\mathrm{oo}}B^I_{pq,rs} = \langle 0_I|(\hat{H}-E_{0,I})\hat{P}\hat{E}_{pq}\hat{E}_{rs}|0_I\rangle = \langle 0_I|\hat{E}_{sr}\hat{E}_{qp}(\hat{H}-E_{0,I})\hat{P}|0_I\rangle^* \tag{S81} $$

where $\langle 0_I|\hat{P}|0_I\rangle$ is normalized to unity. $\mathbf{P}^{IK}$ is the spin-incomplete transition density matrix of ECIS. Using these, it is easy to show that the second derivative of the Ith energy is

$$
\begin{aligned}
{}^{\mathrm{oo}}H^I_{pq,rs} = \mathcal{P}(pq)\mathcal{P}(rs)\Bigg(& {}^{\mathrm{oo}}A^{I*}_{pq,rs} + {}^{\mathrm{oo}}A^I_{pq,rs} + \frac{1}{2}{}^{\mathrm{oo}}B^I_{pq,rs} + \frac{1}{2}{}^{\mathrm{oo}}B^I_{rs,pq} + \frac{1}{2}{}^{\mathrm{oo}}B^{I*}_{pq,rs} + \frac{1}{2}{}^{\mathrm{oo}}B^{I*}_{rs,pq} \\
& - \sum_K \Big((L^{I^*K}_{qp} - \bar{L}^{I^*K}_{qp})(P^{KI}_{sr} - \bar{P}^{KI}_{sr}) + (L^{I^*K}_{sr} - \bar{L}^{I^*K}_{sr})(P^{KI}_{qp} - \bar{P}^{KI}_{qp}) \Big)\Bigg).
\end{aligned} \tag{S82}
$$

For real orbitals,

$$ {}^{\mathrm{oo}}H^I_{pq,rs} = \mathcal{P}(pq)\mathcal{P}(rs)\Bigg(2\,{}^{\mathrm{oo}}A^I_{pq,rs} + {}^{\mathrm{oo}}B^I_{pq,rs} + {}^{\mathrm{oo}}B^I_{rs,pq} - \sum_K \Big((L^{I^*K}_{qp} + L^{KI^*}_{qp})(P^{KI}_{sr} + P^{IK}_{sr}) + (L^{I^*K}_{sr} + L^{KI^*}_{sr})(P^{KI}_{qp} + P^{IK}_{qp}) \Big)\Bigg). \tag{S83} $$

Furthermore, since

$$\hat{E}_{rs}\hat{E}_{pq} = \hat{E}_{pq}\hat{E}_{rs} - \hat{E}_{ps}\delta_{qr} + \hat{E}_{rq}\delta_{ps}, \tag{S84}$$

and thus

$${}^{\mathrm{oo}}B^{I}_{rs,pq} = {}^{\mathrm{oo}}B^{I}_{pq,rs} - L^{I^*I}_{sp}\delta_{qr} + L^{I^*I}_{qr}\delta_{ps}, \tag{S85}$$

we have

$$\begin{aligned}{}^{\mathrm{oo}}H^{I}_{pq,rs} = \mathcal{P}(pq)\mathcal{P}(rs)\Bigg(&2{}^{\mathrm{oo}}A^{I}_{pq,rs} + 2{}^{\mathrm{oo}}B^{I}_{pq,rs} - L^{I^*I}_{sp}\delta_{qr} + L^{I^*I}_{qr}\delta_{ps} \\ &- \sum_K \Big((L^{I^*K}_{qp} + L^{KI^*}_{qp})(P^{KI}_{sr} + P^{IK}_{sr}) + (L^{I^*K}_{sr} + L^{KI^*}_{sr})(P^{KI}_{qp} + P^{IK}_{qp})\Big)\Bigg).\end{aligned} \tag{S86}$$

Using $\hat{R}_g a^\dagger_p \hat{R}^\dagger_g = \sum_q a^\dagger_q (\mathbf{R}_g)_{qp}$,

$$\begin{aligned}{}^{\mathrm{oo}}A^{I}_{qp,rs} &= \sum_g w_g \langle 0_I | a^\dagger_p a_q (\hat{H} - E_{0,I}) \hat{R}_g a^\dagger_r a_s | 0_I \rangle \\ &= \sum_g w_g \langle 0_I | a^\dagger_p a_q (\hat{H} - E_{0,I}) a^\dagger_t a_u \hat{R}_g | 0_I \rangle (\mathbf{R}_g)_{tr} (\mathbf{R}_g)^*_{us} \\ &= \sum_g w_g n_g ({}^{\mathrm{oo}}\mathbf{A}^{I}_g)_{qp,tu} (\mathbf{R}_g)_{tr} (\mathbf{R}_g)^*_{us}\end{aligned} \tag{S87}$$

$$\begin{aligned}{}^{\mathrm{oo}}B^{I}_{pq,rs} &= \sum_g w_g \langle 0_I | (\hat{H} - E_{0,I}) \hat{R}_g a^\dagger_p a_q a^\dagger_r a_s | 0_I \rangle \\ &= \sum_g w_g \langle 0_I | (\hat{H} - E_{0,I}) a^\dagger_t a_u a^\dagger_v a_w \hat{R}_g | 0_I \rangle (\mathbf{R}_g)_{tp} (\mathbf{R}_g)^*_{uq} (\mathbf{R}_g)_{vr} (\mathbf{R}_g)^*_{ws} \\ &= \sum_g w_g n_g ({}^{\mathrm{oo}}\mathbf{B}^{I}_g)_{tu,vw} (\mathbf{R}_g)_{tp} (\mathbf{R}_g)^*_{uq} (\mathbf{R}_g)_{vr} (\mathbf{R}_g)^*_{ws}\end{aligned} \tag{S88}$$

where we have defined

$$({}^{\mathrm{oo}}\mathbf{A}^{I}_g)_{qp,rs} = \frac{\langle 0_I | a^\dagger_p a_q (\hat{H} - E_{0,I}) a^\dagger_r a_s \hat{R}_g | 0_I \rangle}{\langle \Phi_0 | \hat{R}_g | \Phi_0 \rangle} \tag{S89}$$

$$({}^{\mathrm{oo}}\mathbf{B}^{I}_g)_{pq,rs} = \frac{\langle 0_I | (\hat{H} - E_{0,I}) a^\dagger_p a_q a^\dagger_r a_s \hat{R}_g | 0_I \rangle}{\langle \Phi_0 | \hat{R}_g | \Phi_0 \rangle} \tag{S90}$$

Using

$$({}^{\mathrm{oo}}\tilde{\mathbf{A}}^{I}_g)_{qp,rs} = \frac{\langle 0_I | \left[a^\dagger_p a_q, (\hat{H} - E_{0,I}) \right] a^\dagger_r a_s \hat{R}_g | 0_I \rangle}{\langle \Phi_0 | \hat{R}_g | \Phi_0 \rangle} \tag{S91}$$

we can express ${}^{\mathrm{oo}}\mathbf{A}^{I}_g$ as

$$({}^{\mathrm{oo}}\mathbf{A}^{I}_g)_{qp,rs} = (\tilde{\mathbf{A}}^{I}_g)_{qp,rs} + (\mathbf{B}^{I}_g)_{pq,rs} \tag{S92}$$

Now we derive the sigma-vectors ${}^{\mathrm{oo}}\mathbf{A}^{I}\,{}^{\mathrm{o}}\mathbf{x}$ and ${}^{\mathrm{oo}}\mathbf{B}^{I}\,{}^{\mathrm{o}}\mathbf{x}$. We consider

$$\begin{aligned}\sum_{rs} {}^{\mathrm{oo}}A^{I}_{qp,rs}\,{}^{\mathrm{o}}x_{rs} &= \sum_g w_g n_g \sum_{rs} \left(({}^{\tilde{\mathrm{oo}}}\mathbf{A}^{I}_g)_{qp,tu} + ({}^{\mathrm{oo}}\mathbf{B}^{I}_g)_{pq,tu}\right) (\mathbf{R}_g)_{tr} (\mathbf{R}^*_g)_{us}\,{}^{\mathrm{o}}x_{rs} \\ &= \sum_g w_g n_g \sum_{rs} \left(({}^{\mathrm{oo}}\tilde{\mathbf{A}}^{I}_g)_{qp,rs} + ({}^{\mathrm{oo}}\mathbf{B}^{I}_g)_{pq,rs}\right) {}^{\mathrm{o}}\tilde{x}_{rs}\end{aligned} \tag{S93}$$

$$\begin{aligned}\sum_{rs} {}^{\mathrm{oo}}B^{I}_{pq,rs}\,{}^{\mathrm{o}}x_{rs} &= \sum_g w_g n_g \sum_{rstuvw} ({}^{\mathrm{oo}}\mathbf{B}^{I}_g)_{tu,vw} (\mathbf{R}_g)_{tp} (\mathbf{R}_g)^*_{uq} (\mathbf{R}_g)_{vr} (\mathbf{R}_g)^*_{ws}\,{}^{\mathrm{o}}x_{rs} \\ &= \sum_g w_g n_g \sum_{tu} \left(\sum_{rs} ({}^{\mathrm{oo}}\mathbf{B}^{I}_g)_{tu,rs}\,{}^{\mathrm{o}}\tilde{x}_{rs} \right) (\mathbf{R}_g)_{tp} (\mathbf{R}_g)^*_{uq}\end{aligned} \tag{S94}$$

where

$$ {}^{\mathrm{o}}\tilde{x}_{rs} \equiv \sum_{tu} (\mathbf{R}_g)_{rt} (\mathbf{R}_g^*)_{su} {}^{\mathrm{o}}x_{tu} \tag{S95} $$

This result indicates that it suffices to derive the contractions between ${}^{\mathrm{o}}\tilde{x}_{rs}$ and ${}^{\mathrm{oo}}\tilde{\mathbf{A}}_g^I$ and ${}^{\mathrm{oo}}\mathbf{B}_g^I$.

For convenience, we define the following intermediates,

$$ \mathbf{Q}_g^I[{}^{\mathrm{o}}\tilde{\mathbf{x}}] = \boldsymbol{\eta}_g {}^{\mathrm{o}}\tilde{\mathbf{x}} \boldsymbol{\gamma}_g^I - \boldsymbol{\gamma}_g^{I\,\mathrm{o}}\tilde{\mathbf{x}} \boldsymbol{\rho}_g - \boldsymbol{\mathcal{D}}_g^{I\,\mathrm{o}}\tilde{\mathbf{x}} \boldsymbol{\mathcal{C}}_g^I - \boldsymbol{\mathcal{C}}_g^{I\,\mathrm{o}}\tilde{\mathbf{x}} \boldsymbol{\mathcal{D}}_g^I + \mathrm{Tr}[\boldsymbol{\mathcal{D}}_g^{I\,\mathrm{o}}\tilde{\mathbf{x}}] \boldsymbol{\mathcal{C}}_g^I + \mathrm{Tr}[\boldsymbol{\mathcal{C}}_g^{I\,\mathrm{o}}\tilde{\mathbf{x}}] \boldsymbol{\mathcal{D}}_g^I \tag{S96} $$

$$ \bar{\boldsymbol{\mathcal{C}}}_g^I[{}^{\mathrm{o}}\tilde{\mathbf{x}}] = \boldsymbol{\eta}_g {}^{\mathrm{o}}\tilde{\mathbf{x}} \boldsymbol{\mathcal{C}}_g^I - \boldsymbol{\mathcal{C}}_g^{I\,\mathrm{o}}\tilde{\mathbf{x}} \boldsymbol{\rho}_g \tag{S97} $$

$$ \bar{\boldsymbol{\mathcal{D}}}_g^I[{}^{\mathrm{o}}\tilde{\mathbf{x}}] = \boldsymbol{\eta}_g {}^{\mathrm{o}}\tilde{\mathbf{x}} \boldsymbol{\mathcal{D}}_g^I - \boldsymbol{\mathcal{D}}_g^{I\,\mathrm{o}}\tilde{\mathbf{x}} \boldsymbol{\rho}_g \tag{S98} $$

$$ \mathbf{X}_g[{}^{\mathrm{o}}\tilde{\mathbf{x}}] = \boldsymbol{\eta}_g {}^{\mathrm{o}}\tilde{\mathbf{x}} \boldsymbol{\rho}_g \tag{S99} $$

and let

$$ \boldsymbol{\Omega}_g^I = \mathbf{Q}_g^I[{}^{\mathrm{o}}\tilde{\mathbf{x}}] + \mathbf{X}_g N_g^I + \boldsymbol{\gamma}_g^{II} \mathrm{Tr}[\boldsymbol{\rho}_g {}^{\mathrm{o}}\tilde{\mathbf{x}}] \tag{S100} $$

Then, after simple algebra,

$$ \begin{aligned} \sum_{rs} ({}^{\mathrm{oo}}\tilde{\mathbf{A}}_g^I)_{qp,rs} {}^{\mathrm{o}}\tilde{x}_{rs} = \Bigg(& \Big[\mathbf{F}_g, \boldsymbol{\Omega}_g^I\Big] + \Big[\mathbf{G}[\boldsymbol{\Omega}_g^I], \boldsymbol{\rho}_g\Big] + \Big[\mathbf{F}_g, \boldsymbol{\rho}_g\Big] \mathrm{Tr}[\mathbf{P}_g^{II\,\mathrm{o}}\tilde{\mathbf{x}}] \\ & + \Big[\mathbf{G}[\boldsymbol{\mathcal{D}}_g^I], \bar{\boldsymbol{\mathcal{C}}}_g^I\Big] + \Big[\mathbf{G}[\boldsymbol{\mathcal{C}}_g^I], \bar{\boldsymbol{\mathcal{D}}}_g^I\Big] + \Big[\mathbf{G}[\bar{\boldsymbol{\mathcal{C}}}_g^I], \boldsymbol{\mathcal{D}}_g^I\Big] + \Big[\mathbf{G}[\bar{\boldsymbol{\mathcal{D}}}_g^I], \boldsymbol{\mathcal{C}}_g^I\Big] \\ & + \big[\mathbf{G}[\boldsymbol{\gamma}_g^I], \mathbf{X}_g\big] + \big[\mathbf{G}[\mathbf{X}_g], \boldsymbol{\gamma}_g^I\big] + \Big(\Big[\mathbf{G}[\boldsymbol{\mathcal{D}}_g^I], \boldsymbol{\mathcal{C}}_g^I\Big] + \Big[\mathbf{G}[\boldsymbol{\mathcal{C}}_g^I], \boldsymbol{\mathcal{D}}_g^I\Big]\Big) \mathrm{Tr}[\boldsymbol{\rho}_g {}^{\mathrm{o}}\tilde{\mathbf{x}}] \Bigg)_{qp} \end{aligned} \tag{S101} $$

$$ \begin{aligned} \sum_{rs} ({}^{\mathrm{oo}}\mathbf{B}_g^I)_{pq,rs} {}^{\mathrm{o}}\tilde{x}_{rs} = \Bigg(& (H_g^I - E_{0,I} N_g^I)(\boldsymbol{\rho}_g {}^{\mathrm{o}}\tilde{\mathbf{x}} \boldsymbol{\rho}_g - \mathrm{Tr}[\boldsymbol{\rho}_g {}^{\mathrm{o}}\tilde{\mathbf{x}}] \boldsymbol{\rho}_g) + \boldsymbol{\eta}_g {}^{\mathrm{o}}\tilde{\mathbf{x}} \mathbf{L}_g^I - \mathbf{L}_g^{I\,\mathrm{o}}\tilde{\mathbf{x}} \boldsymbol{\rho}_g + \mathbf{L}_g^I \mathrm{Tr}[\boldsymbol{\rho}_g {}^{\mathrm{o}}\tilde{\mathbf{x}}] + \boldsymbol{\rho}_g \mathrm{Tr}[\mathbf{L}_g^{I\,\mathrm{o}}\tilde{\mathbf{x}}] \\ & + (\epsilon_g - E_{0,I})\Big(\mathbf{Q}_g^I - \boldsymbol{\eta}_g {}^{\mathrm{o}}\tilde{\mathbf{x}} \boldsymbol{\gamma}_g^{II} + \boldsymbol{\gamma}_g^{II\,\mathrm{o}}\tilde{\mathbf{x}} \boldsymbol{\rho}_g\Big) + \Big(\mathbf{Q}_g^I - \boldsymbol{\eta}_g {}^{\mathrm{o}}\tilde{\mathbf{x}} \boldsymbol{\gamma}_g^{II}\Big) \mathbf{F}_g \boldsymbol{\eta}_g - \boldsymbol{\rho}_g \mathbf{F}_g \Big(\mathbf{Q}_g^I + \boldsymbol{\gamma}_g^{II\,\mathrm{o}}\tilde{\mathbf{x}} \boldsymbol{\rho}_g\Big) \\ & + \boldsymbol{\mathcal{C}}_g^I({}^{\mathrm{o}}\tilde{\mathbf{x}} \boldsymbol{\rho}_g \mathbf{F}_g - \mathbf{F}_g \boldsymbol{\eta}_g {}^{\mathrm{o}}\tilde{\mathbf{x}}) \boldsymbol{\mathcal{D}}_g^I + \boldsymbol{\mathcal{D}}_g^I({}^{\mathrm{o}}\tilde{\mathbf{x}} \boldsymbol{\rho}_g \mathbf{F}_g - \mathbf{F}_g \boldsymbol{\eta}_g {}^{\mathrm{o}}\tilde{\mathbf{x}}) \boldsymbol{\mathcal{C}}_g^I + \boldsymbol{\rho}_g \mathbf{F}_g \boldsymbol{\eta}_g \mathrm{Tr}[\boldsymbol{\gamma}_g^{II\,\mathrm{o}}\tilde{\mathbf{x}}] \\ & - \boldsymbol{\rho}_g \mathbf{G}[\boldsymbol{\mathcal{C}}_g^I] \boldsymbol{\eta}_g {}^{\mathrm{o}}\tilde{\mathbf{x}} \boldsymbol{\mathcal{D}}_g^I - \boldsymbol{\mathcal{D}}_g^{I\,\mathrm{o}}\tilde{\mathbf{x}} \boldsymbol{\rho}_g \mathbf{G}[\boldsymbol{\mathcal{C}}_g^I] \boldsymbol{\eta}_g - \boldsymbol{\rho}_g \mathbf{G}[\boldsymbol{\mathcal{D}}_g^I] \boldsymbol{\eta}_g {}^{\mathrm{o}}\tilde{\mathbf{x}} \boldsymbol{\mathcal{C}}_g^I - \boldsymbol{\mathcal{C}}_g^{I\,\mathrm{o}}\tilde{\mathbf{x}} \boldsymbol{\rho}_g \mathbf{G}[\boldsymbol{\mathcal{D}}_g^I] \boldsymbol{\eta}_g \\ & + \boldsymbol{\mathcal{C}}_g^I \mathrm{Tr}[\mathbf{F}_g \bar{\boldsymbol{\mathcal{D}}}_g^I] + \boldsymbol{\mathcal{D}}_g^I \mathrm{Tr}[\mathbf{F}_g \bar{\boldsymbol{\mathcal{C}}}_g^I] + \boldsymbol{\mathcal{D}}_g^I \mathbf{G}[\bar{\boldsymbol{\mathcal{C}}}_g^I] \boldsymbol{\eta}_g - \boldsymbol{\rho}_g \mathbf{G}[\bar{\boldsymbol{\mathcal{C}}}_g^I] \boldsymbol{\mathcal{D}}_g^I + \boldsymbol{\mathcal{C}}_g^I \mathbf{G}[\bar{\boldsymbol{\mathcal{D}}}_g^I] \boldsymbol{\eta}_g - \boldsymbol{\rho}_g \mathbf{G}[\bar{\boldsymbol{\mathcal{D}}}_g^I] \boldsymbol{\mathcal{C}}_g^I \\ & + \boldsymbol{\mathcal{C}}_g^I \mathrm{Tr}\Big(\mathbf{G}[\mathbf{X}_g] \boldsymbol{\mathcal{D}}_g^I\Big) - \boldsymbol{\mathcal{C}}_g^I \mathbf{G}[\mathbf{X}_g] \boldsymbol{\mathcal{D}}_g + \boldsymbol{\mathcal{D}}_g^I \mathrm{Tr}\Big(\mathbf{G}[\mathbf{X}_g] \boldsymbol{\mathcal{C}}_g^I\Big) - \boldsymbol{\mathcal{D}}_g^I \mathbf{G}[\mathbf{X}_g] \boldsymbol{\mathcal{C}}_g^I \\ & + \boldsymbol{\gamma}_g^{II} \mathrm{Tr}[\mathbf{F}_g \mathbf{X}_g] + \mathbf{P}_g^I \mathbf{G}[\mathbf{X}_g] \boldsymbol{\eta}_g - \boldsymbol{\rho}_g \mathbf{G}[\mathbf{X}_g] \boldsymbol{\gamma}_g^{II} + \boldsymbol{\rho}_g \mathbf{G}[\mathbf{Q}_g^I] \boldsymbol{\eta}_g \Bigg)_{qp} \end{aligned} \tag{S102} $$

With these, one can evaluate the contraction of $\sum_{bj} {}^{\mathrm{oo}}H_{ai,bj} {}^{\mathrm{o}}x_{bj}$.

B. Contraction of ${}^{\mathrm{oc}}H_{ai,\mu}^I$ with ${}^{\mathrm{c}}x_\mu^I$ and ${}^{\mathrm{o}}x_{ai}$

We have two types of sigma-vectors. The first one is contracted with the CI trial vector ${}^{\mathrm{c}}\mathbf{x}^I$. We conveniently define the following quantities:

$$ |\mathbf{x}^I\rangle = \sum_\mu {}^{\mathrm{c}}x_\mu^I |\Phi_\mu\rangle \tag{S103} $$

and

$$ L_{pq}^{I^*\mathbf{x}} = \langle 0_I | \hat{E}_{qp} (\hat{H} - E_{0,I}) \hat{P} | \mathbf{x}^I \rangle = \sum_\mu {}^{\mathrm{oc}}A_{pq,\mu}^I {}^{\mathrm{c}}x_\mu^I \tag{S104} $$

$$ L_{pq}^{\mathbf{x}I^*} = \langle \mathbf{x}^I | \hat{E}_{qp} (\hat{H} - E_{0,I}) \hat{P} | 0_I \rangle = \sum_\mu {}^{\mathrm{co}}B_{\mu,pq}^I {}^{\mathrm{c}}x_\mu^I \tag{S105} $$

$$ \bar{L}_{pq}^{I^*\mathbf{x}} = \langle 0_I | (\hat{H} - E_{0,I}) \hat{P} \hat{E}_{qp} | \mathbf{x}^I \rangle = \sum_\mu {}^{\mathrm{oc}}B_{pq,\mu}^I {}^{\mathrm{c}}x_\mu^I = (L_{qp}^{\mathbf{x}I^*})^* \tag{S106} $$

$$ \bar{L}_{pq}^{\mathbf{x}I^*} = \langle \mathbf{x}^I | (\hat{H} - E_{0,I}) \hat{P} \hat{E}_{qp} | 0_I \rangle = \sum_\mu {}^{\mathrm{co}}A_{\mu,pq}^I {}^{\mathrm{c}}x_\mu^I = (L_{qp}^{I^*\mathbf{x}})^* \tag{S107} $$

where, again, the star indicates the state for which the energy $E_{0,I}$ is used. $\bar{L}_{pq}^{I^*\mathbf{x}}$ can be obtained in exactly the same manner, by simply replacing $\mathbf{c}^K$ by ${}^{\mathrm{c}}\mathbf{x}^I$ in $\bar{\mathbf{L}}^{I^*K}$.

Then, the sigma-vector contracted with ${}^{\mathrm{c}}x_\mu^I$ is

$$\sum_\mu {}^{\mathrm{oc}}H_{pq,\mu}^I \, {}^{\mathrm{c}}x_\mu^I = 2\mathcal{P}(pq)\left(\frac{1}{n}\Big(L_{qp}^{I^*\mathbf{x}} + L_{qp}^{\mathbf{x}I^*} - \sum_{K=1}^{n}(L_{qp}^{I^*K} + L_{qp}^{KI^*})\langle 0_K|\hat{P}|\mathbf{x}^I\rangle\Big) - \sum_\mu \left({}^{\mathrm{c}}g_\mu^{I\,\mathrm{c}}x_\mu^I\right) P_{qp}^I\right) \tag{S108}$$

where we have used the fact all the quantities are real.

The second sigma-vector is the one contracted with ${}^{\mathrm{o}}x_{ai}$.

$$\begin{aligned}\sum_{pq} {}^{\mathrm{co}}H_{\mu,pq}^I \, {}^{\mathrm{o}}x_{pq} = -\frac{4}{n}\sum_\mu \Bigg(&\langle \Phi_\mu|(\hat{H} - E_{0,I})\hat{P}\hat{E}_{pq}|0_I\rangle^{\mathrm{o}}x_{pq} + \langle 0_I|(\hat{H} - E_{0,I})\hat{P}\hat{E}_{pq}|\Phi_\mu\rangle^{\mathrm{o}}x_{pq} \\ &+ \sum_{K=1}^{n} \mathrm{Tr}\left[(\mathbf{L}^{KI^*} + \mathbf{L}^{I^*K})^{\mathrm{o}}\mathbf{x}\right]\langle 0_K|\hat{P}|\Phi_\mu\rangle + \mathrm{Tr}[\mathbf{P}^{I\,\mathrm{o}}\mathbf{x}]^{\mathrm{c}}g_\mu^I\Bigg)\end{aligned} \tag{S109}$$

We can extract the matrix elements ${}^{\mathrm{co}}A_{\mu,pq}^I = \langle \Phi_\mu|(\hat{H} - E_{0,I})\hat{P}\hat{E}_{pq}|0_I\rangle$ and ${}^{\mathrm{oc}}B_{pq,\mu}^I = \langle 0_I|(\hat{H} - E_{0,I})\hat{P}\hat{E}_{pq}|\Phi_\mu\rangle$ by taking the derivatives of $L_{qp}^{KI^*}$ and $L_{qp}^{I^*K}$ with respect to the CI coefficients c_μ^K. The result is

$$\begin{aligned}\sum_{pq} {}^{\mathrm{co}}A_{0,pq}^I \, {}^{\mathrm{o}}x_{pq} &= \sum_{pq}\langle \Phi_0|(\hat{H} - E_{0,I})\hat{P}\hat{E}_{pq}|0_I\rangle^{\mathrm{o}}x_{pq} \\ &= \sum_g w_g n_g\Big(\mathrm{Tr}[\boldsymbol{\rho}_g{}^{\mathrm{o}}\tilde{\mathbf{x}}]\Big((\epsilon_g - E_{0,I})\tilde{c}_0 + \mathrm{Tr}[\mathbf{F}_g\boldsymbol{\mathcal{C}}_g^I]\Big) + \mathrm{Tr}[\boldsymbol{\mathcal{C}}_g^{I\,\mathrm{o}}\tilde{\mathbf{x}}](\epsilon_g - E_{0,I}) - \mathrm{Tr}[\boldsymbol{\mathcal{C}}_g^{I\,\mathrm{o}}\tilde{\mathbf{x}}\boldsymbol{\rho}_g\mathbf{F}_g] \\ &+ \mathrm{Tr}[\boldsymbol{\mathcal{C}}_g^I\mathbf{F}_g\boldsymbol{\eta}_g{}^{\mathrm{o}}\tilde{\mathbf{x}}] + \mathrm{Tr}[\mathbf{F}_g\mathbf{X}_g]\tilde{c}_0^I + \mathrm{Tr}[\mathbf{G}[\boldsymbol{\mathcal{C}}_g^I]\mathbf{X}_g]\Big)\end{aligned} \tag{S110}$$

$$\begin{aligned}\sum_{pq} {}^{\mathrm{oc}}B_{pq,0}^I \, {}^{\mathrm{o}}x_{pq} &= \sum_{pq}\langle 0_I|(\hat{H} - E_{0,I})\hat{P}\hat{E}_{pq}|\Phi_0\rangle^{\mathrm{o}}x_{pq} \\ &= \sum_g w_g n_g\Big(\mathrm{Tr}[\boldsymbol{\rho}_g{}^{\mathrm{o}}\tilde{\mathbf{x}}]\Big((\epsilon_g - E_{0,I})\tilde{d}_0 + \mathrm{Tr}[\mathbf{F}_g\boldsymbol{\mathcal{D}}_g^I]\Big) + \mathrm{Tr}[\boldsymbol{\mathcal{D}}_g^{I\,\mathrm{o}}\tilde{\mathbf{x}}](\epsilon_g - E_{0,I}) - \mathrm{Tr}[\boldsymbol{\mathcal{D}}_g^{I\,\mathrm{o}}\tilde{\mathbf{x}}\boldsymbol{\rho}_g\mathbf{F}_g] \\ &+ \mathrm{Tr}[\boldsymbol{\mathcal{D}}_g^I\mathbf{F}_g\boldsymbol{\eta}_g{}^{\mathrm{o}}\tilde{\mathbf{x}}] + \mathrm{Tr}[\mathbf{F}_g\mathbf{X}_g]\tilde{d}_0^I + \mathrm{Tr}[\mathbf{G}[\boldsymbol{\mathcal{D}}_g^I]\mathbf{X}_g]\Big)\end{aligned} \tag{S111}$$

which are symmetric with respect to $\tilde{c}_0, \boldsymbol{\mathcal{C}}_g^I$ and $\tilde{d}_0, \boldsymbol{\mathcal{D}}_g^I$. We als have

$$\begin{aligned}\sum_{pq} {}^{\mathrm{co}}A_{ai,pq}^I \, {}^{\mathrm{o}}x_{pq} &= \sum_{pq}\langle \Phi_i^a|(\hat{H} - E_{0,I})\hat{P}\hat{E}_{pq}|0_I\rangle^{\mathrm{o}}x_{pq} \\ &= \sum_g w_g n_g\Big(\mathrm{Tr}[\boldsymbol{\rho}_g{}^{\mathrm{o}}\tilde{\mathbf{x}}]\Big((\epsilon_g - E_{0,I})(\tilde{c}_0^I\boldsymbol{\mathcal{W}}_g + \boldsymbol{\mathcal{C}}_g^I) + \tilde{c}_0^I\boldsymbol{\mathcal{L}}_g\mathbf{F}_g\boldsymbol{\mathcal{R}}_g + \mathrm{Tr}[\mathbf{F}_g\boldsymbol{\mathcal{C}}_g^I]\boldsymbol{\mathcal{W}}_g \\ &+ \boldsymbol{\mathcal{L}}_g\mathbf{F}_g\boldsymbol{\mathcal{C}}_g^I - \boldsymbol{\mathcal{C}}_g^I\mathbf{F}_g\boldsymbol{\mathcal{R}}_g + \boldsymbol{\mathcal{L}}_g\mathbf{G}[\boldsymbol{\mathcal{C}}_g^I]\boldsymbol{\mathcal{R}}_g\Big) \\ &+ \tilde{c}_0^I\boldsymbol{\mathcal{L}}_g\mathbf{T}_g\boldsymbol{\mathcal{R}}_g + \mathrm{Tr}[\boldsymbol{\mathcal{C}}_g^I\mathbf{T}_g]\boldsymbol{\mathcal{W}}_g + \boldsymbol{\mathcal{L}}_g\mathbf{T}_g\boldsymbol{\mathcal{C}}_g^I - \boldsymbol{\mathcal{C}}_g^I\mathbf{T}_g\boldsymbol{\mathcal{R}}_g + \mathrm{Tr}[\mathbf{F}_g\mathbf{X}_g](\tilde{c}_0^I\boldsymbol{\mathcal{W}}_g + \boldsymbol{\mathcal{C}}_g^I) \\ &+ \boldsymbol{\mathcal{L}}_g{}^{\mathrm{o}}\tilde{\mathbf{x}}\boldsymbol{\mathcal{R}}_g\mathrm{Tr}[\mathbf{F}_g\boldsymbol{\mathcal{C}}_g^I] - \boldsymbol{\mathcal{L}}_g\mathbf{F}_g\boldsymbol{\mathcal{C}}_g^{I\,\mathrm{o}}\tilde{\mathbf{x}}\boldsymbol{\mathcal{R}}_g - \boldsymbol{\mathcal{L}}_g{}^{\mathrm{o}}\tilde{\mathbf{x}}\boldsymbol{\mathcal{C}}_g^I\mathbf{F}_g\boldsymbol{\mathcal{R}}_g + \mathrm{Tr}[\boldsymbol{\mathcal{C}}_g^{I\,\mathrm{o}}\tilde{\mathbf{x}}]\boldsymbol{\mathcal{L}}_g\mathbf{F}_g\boldsymbol{\mathcal{R}}_g \\ &+ \boldsymbol{\mathcal{L}}_g\mathbf{G}[\tilde{\boldsymbol{\mathcal{C}}}_g{}^I]\boldsymbol{\mathcal{R}}_g + \boldsymbol{\mathcal{L}}_g\mathbf{G}[\boldsymbol{\mathcal{C}}_g^I]\boldsymbol{\eta}_g{}^{\mathrm{o}}\tilde{\mathbf{x}}\boldsymbol{\mathcal{R}}_g - \boldsymbol{\mathcal{L}}_g{}^{\mathrm{o}}\tilde{\mathbf{x}}\boldsymbol{\rho}_g\mathbf{G}[\boldsymbol{\mathcal{C}}_g^I]\boldsymbol{\mathcal{R}}_g\Big)_{ai}\end{aligned} \tag{S112}$$

and

$$\sum_{pq} {}^{\mathrm{oc}}B^I_{pq,ck}\, {}^{\mathrm{o}}x_{pq} = \sum_{pq} \langle 0_I|(\hat{H}-E_{0,I})\hat{P}\hat{E}_{pq}|\Phi_k^c\rangle {}^{\mathrm{o}}x_{pq}$$
$$= \sum_g w_g n_g \Bigg[\mathrm{Tr}[\boldsymbol{\rho}_g {}^{\mathrm{o}}\tilde{\mathbf{x}}]\Big((\epsilon_g - E_{0,I})(\tilde{d}_0^I \boldsymbol{\mathcal{W}}_g + \boldsymbol{\mathcal{D}}_g^I)_{kc} + \tilde{d}_0^I(\boldsymbol{\mathcal{L}}_g\mathbf{F}_g\boldsymbol{\mathcal{R}}_g)_{kc} + \mathrm{Tr}[\mathbf{F}_g\boldsymbol{\mathcal{D}}_g^I](\boldsymbol{\mathcal{W}}_g)_{kc}$$
$$+ c_{ai}^{I*}(\boldsymbol{\mathcal{L}}_g\mathbf{F}_g\boldsymbol{\mathcal{R}}_g)_{ac}(\boldsymbol{\mathcal{W}}_g)_{ki} - c_{ai}^{I*}(\boldsymbol{\mathcal{W}}_g)_{ac}(\boldsymbol{\mathcal{L}}_g\mathbf{F}_g\boldsymbol{\mathcal{R}}_g)_{ki} + (\boldsymbol{\mathcal{L}}_g\mathbf{G}[\boldsymbol{\mathcal{D}}_g]\boldsymbol{\mathcal{R}}_g)_{kc}\Big)$$
$$+ \tilde{d}_0^I\boldsymbol{\mathcal{L}}_g\mathbf{T}_g\boldsymbol{\mathcal{R}}_g + \mathrm{Tr}[\boldsymbol{\mathcal{D}}_g^I\mathbf{T}_g](\boldsymbol{\mathcal{W}}_g)_{kc} + (\boldsymbol{\mathcal{W}}_g)_{ki}c_{ai}^*(\boldsymbol{\mathcal{L}}_g\mathbf{T}_g\boldsymbol{\mathcal{R}}_g)_{ac} - c_{ai}^*(\boldsymbol{\mathcal{W}}_g)_{ac}(\boldsymbol{\mathcal{L}}_g\mathbf{T}_g\boldsymbol{\mathcal{R}}_g)_{ki}$$
$$+ \mathrm{Tr}[\mathbf{F}_g\mathbf{X}_g]\Big(\tilde{d}_0(\boldsymbol{\mathcal{W}}_g)_{kc} + c_{ai}^*(\boldsymbol{\mathcal{W}}_g)_{ac}(\boldsymbol{\mathcal{W}}_g)_{ki}\Big)$$
$$+ (\boldsymbol{\mathcal{L}}_g{}^{\mathrm{o}}\tilde{\mathbf{x}}\boldsymbol{\mathcal{R}}_g)_{kc}\mathrm{Tr}[\mathbf{F}_g\boldsymbol{\mathcal{D}}_g] - (\boldsymbol{\mathcal{L}}_g{}^{\mathrm{o}}\tilde{\mathbf{x}}\boldsymbol{\mathcal{D}}_g\mathbf{F}_g\boldsymbol{\mathcal{R}}_g)_{kc} - (\boldsymbol{\mathcal{L}}_g\mathbf{F}_g\boldsymbol{\mathcal{D}}_g{}^{\mathrm{o}}\tilde{\mathbf{x}}\boldsymbol{\mathcal{R}}_g)_{kc} + \mathrm{Tr}[\boldsymbol{\mathcal{D}}_g{}^{\mathrm{o}}\tilde{\mathbf{x}}](\boldsymbol{\mathcal{L}}_g\mathbf{F}_g\boldsymbol{\mathcal{R}}_g)_{kc}$$
$$+ (\boldsymbol{\mathcal{L}}_g\mathbf{G}[\bar{\boldsymbol{\mathcal{D}}}_g]\boldsymbol{\mathcal{R}}_g)_{kc} + (\boldsymbol{\mathcal{L}}_g\mathbf{G}[\boldsymbol{\mathcal{D}}_g]\boldsymbol{\eta}_g{}^{\mathrm{o}}\tilde{\mathbf{x}}\boldsymbol{\mathcal{R}}_g)_{kc} - (\boldsymbol{\mathcal{L}}_g{}^{\mathrm{o}}\tilde{\mathbf{x}}\boldsymbol{\rho}_g\mathbf{G}[\boldsymbol{\mathcal{D}}_g]\boldsymbol{\mathcal{R}}_g)_{kc}\Bigg] \tag{S113}$$

where

$$\mathbf{T}_g = (\epsilon_g - E_{0,I}){}^{\mathrm{o}}\tilde{\mathbf{x}} + \mathbf{F}_g\boldsymbol{\eta}_g{}^{\mathrm{o}}\tilde{\mathbf{x}} - {}^{\mathrm{o}}\tilde{\mathbf{x}}\boldsymbol{\rho}_g\mathbf{F}_g + \mathbf{G}[\mathbf{X}_g] \tag{S114}$$

C. Contraction of ${}^{\mathrm{cc}}H^{IJ}_{\mu\nu}$ with ${}^{\mathrm{c}}x^I_\nu$

Using Eq.(S103),

$$\sum_\nu {}^{\mathrm{cc}}H^{IJ\mathrm{c}}_{\mu\nu}x^I_\nu = \frac{\delta_{IJ}}{n}\sum_\nu\Bigg(\langle\Phi_\mu|(\hat{H}-E_{0,I})\hat{P}|\Phi_\nu\rangle^{\mathrm{c}}x^I_\nu - \sum_K^n\langle\Phi_\mu|\hat{P}|0_K\rangle\langle 0_K|(\hat{H}-E_{0,K})\hat{P}|\Phi_\nu\rangle^{\mathrm{c}}x^I_\nu$$
$$- \sum_K^n\langle\Phi_\mu|(\hat{H}-E_{0,I})\hat{P}|0_K\rangle\langle 0_K|\hat{P}|\Phi_\nu\rangle^{\mathrm{c}}x^I_\nu + \langle\Phi_\nu|(\hat{H}-E_{0,I})\hat{P}|\Phi_\mu\rangle^{\mathrm{c}}x^I_\nu$$
$$- \sum_K^n\langle\Phi_\nu|\hat{P}|0_K\rangle\langle 0_K|(\hat{H}-E_{0,K})\hat{P}|\Phi_\mu\rangle^{\mathrm{c}}x^I_\nu - \sum_K^n\langle\Phi_\nu|(\hat{H}-E_{0,I})\hat{P}|0_K\rangle\langle 0_K|\hat{P}|\Phi_\mu\rangle^{\mathrm{c}}x^I_\nu\Bigg)$$
$$= \frac{\delta_{IJ}}{n}\Bigg(\langle\Phi_\mu|(\hat{H}-E_{0,I})\hat{P}|\mathbf{x}_I\rangle - \sum_K^n\langle\Phi_\mu|\hat{P}|0_K\rangle\langle 0_K|(\hat{H}-E_{0,K})\hat{P}|\mathbf{x}_I\rangle - \sum_K^n\langle\Phi_\mu|(\hat{H}-E_{0,I})\hat{P}|0_K\rangle\langle 0_K|\hat{P}|\mathbf{x}_I\rangle$$
$$+ \langle\mathbf{x}_I|(\hat{H}-E_{0,I})\hat{P}|\Phi_\mu\rangle - \sum_K^n\langle\mathbf{x}_I|\hat{P}|0_K\rangle\langle 0_K|(\hat{H}-E_{0,K})\hat{P}|\Phi_\mu\rangle - \sum_K^n\langle\mathbf{x}_I|(\hat{H}-E_{0,I})\hat{P}|0_K\rangle\langle 0_K|\hat{P}|\Phi_\mu\rangle\Bigg) \tag{S115}$$

which can be evaluated easily.

S5. Double CIS

A. Working equation for spin-projected DCIS (EDCIS)

In the main text, we have expressed the DCIS ansatz as

$$|\Psi_{\mathrm{DCIS}}\rangle = \sum_{ai}{}^{\mathrm{o}}d_{ai}\hat{E}_{ai}|0\rangle + \sum_\mu {}^{\mathrm{c}}d_\mu|\Phi_\mu\rangle \tag{S116}$$

The projected energy is

$$E_{\mathrm{EDCIS}} = \frac{\langle\Psi_{\mathrm{DCIS}}|\hat{H}\hat{P}|\Psi_{\mathrm{DCIS}}\rangle}{\langle\Psi_{\mathrm{DCIS}}|\hat{P}|\Psi_{\mathrm{DCIS}}\rangle} \tag{S117}$$

Taking the derivative of E_{EDCIS}, we find

$$\begin{aligned}\langle 0|\hat{E}_{kc}(\hat{H}-E_{\text{EDCIS}})\hat{P}|\Psi_{\text{DCIS}}\rangle &= \sum_{ck}\langle 0|\hat{E}_{ia}(\hat{H}-E_{\text{EDCIS}})\hat{P}\hat{E}_{ck}|0\rangle^{\text{o}}d_{ck} + \sum_{\nu}\langle 0|\hat{E}_{ia}(\hat{H}-E_{\text{EDCIS}})\hat{P}|\Phi_\nu\rangle^{\text{c}}d_\nu \\ &= \sum_{ck}({}^{\text{oo}}A_{ai,ck} - \omega^{\text{oo}}S_{ai,ck})^{\text{o}}d_{ck} + \sum_{\nu}({}^{\text{oc}}A_{ai,\nu} - \omega^{\text{oc}}S_{ai,\nu})^{\text{c}}d_\nu \\ &= 0 \end{aligned} \tag{S118}$$

$$\begin{aligned}\langle \Phi_\mu|(\hat{H}-E_{\text{EDCIS}})\hat{P}|\Psi_{\text{DCIS}}\rangle &= \sum_{ck}\langle \Phi_\mu|(\hat{H}-E_{\text{EDCIS}})\hat{P}\hat{E}_{ck}|0\rangle^{\text{o}}d_{ck} + \sum_{\nu}\langle \Phi_\mu|(\hat{H}-E_{\text{EDCIS}})\hat{P}|\Phi_\nu\rangle^{\text{c}}d_\nu \\ &= \sum_{ck}({}^{\text{co}}A_{\mu,ck} - \omega^{\text{co}}S_{\mu,ck})^{\text{o}}d_{ck} + \sum_{\nu}({}^{\text{cc}}A_{\mu\nu} - \omega^{\text{cc}}S_{\mu\nu})^{\text{c}}d_\nu \\ &= 0 \end{aligned} \tag{S119}$$

where we remind that

$${}^{\text{oo}}A_{ai,ck} = \langle 0|\hat{E}_{ia}(\hat{H}-E_0)\hat{P}\hat{E}_{ck}|0\rangle \tag{S120}$$

and therefore it is easy to see that

$$\omega = E_{\text{EDCIS}} - E_0 \tag{S121}$$

is the EDCIS relaxation energy from ECIS. Other A matrices can be similarly defined as

$${}^{\text{oc}}A_{ai,\nu} = \langle 0|\hat{E}_{ia}(\hat{H}-E_0)\hat{P}|\Phi_\nu\rangle \tag{S122}$$

$${}^{\text{co}}A_{\mu,ck} = \langle \Phi_\mu|(\hat{H}-E_0)\hat{P}\hat{E}_{ck}|0\rangle \tag{S123}$$

$${}^{\text{cc}}A_{\mu\nu} = \langle \Phi_\mu|(\hat{H}-E_0)\hat{P}|\Phi_\nu\rangle \tag{S124}$$

$$\tag{S125}$$

which are also part of the ECIS Hessian matrix elements. Due to the nonorthogonal nature, the overlap matrix $\mathbf{S}$ was introduced as

$${}^{\text{oo}}S_{ai,ck} = \langle 0|\hat{E}_{ia}\hat{P}\hat{E}_{ck}|0\rangle \tag{S126}$$

$${}^{\text{oc}}S_{ai,\nu} = \langle 0|\hat{E}_{ia}\hat{P}|\Phi_\nu\rangle \tag{S127}$$

$${}^{\text{cc}}S_{\mu\nu} = \langle \Phi_\mu|\hat{P}|\Phi_\nu\rangle \tag{S128}$$

Eqs.(S118) and (S119) can be recast as the following generalized eigenvalue problem:

$$\begin{pmatrix} {}^{\text{oo}}\mathbf{A} & {}^{\text{oc}}\mathbf{A} \\ {}^{\text{co}}\mathbf{A} & {}^{\text{cc}}\mathbf{A} \end{pmatrix} \begin{pmatrix} {}^{\text{o}}\mathbf{d} \\ {}^{\text{c}}\mathbf{d} \end{pmatrix} = \omega \begin{pmatrix} {}^{\text{oo}}\mathbf{S} & {}^{\text{oc}}\mathbf{S} \\ {}^{\text{co}}\mathbf{S} & {}^{\text{cc}}\mathbf{S} \end{pmatrix} \begin{pmatrix} {}^{\text{o}}\mathbf{d} \\ {}^{\text{c}}\mathbf{d} \end{pmatrix} \tag{S129}$$

S6. Simplified expressions for non-projected SA-CIS and DCIS

Having derived all the relevant terms with the spin-projection operator $\hat{P}$, here we summarize the simplified results for non-projected SA-CIS and DCIS by requiring $\hat{P}, \hat{R}_g \to 1$.

A. Energy

Let $c_{ai} = (\mathbf{c})_{ai}$ be the CIS coefficients. Using the normalization condition

$$\langle 0|0\rangle \equiv |c_0|^2 + c_{ai}c^*_{ai} = 1 \tag{S130}$$

and the CIS contribution to the density matrix

$$\gamma_{ji} = -c^*_{bj}c_{bi} \tag{S131}$$

$$\gamma_{ai} = c^*_0 c_{ai} \tag{S132}$$

$$\gamma_{ia} = c_0 c^*_{ai} \tag{S133}$$

$$\gamma_{ab} = c_{aj}c^*_{bj} \tag{S134}$$

it is easy to verify that

$$E_0 = E_{\text{HF}} + (F_{pq}\gamma_{qp}) + G[\mathbf{c}]_{ai}c^*_{ai} \tag{S135}$$

and if we use the canonical orbital basis,

$$E_0 = E_{\rm HF} + [\epsilon_i \gamma_{ii} + \epsilon_b \gamma_{bb}] + G[\mathbf{c}]_{ai} c^*_{ai} \tag{S136}$$

Below, we will derive all the contractions necessary to construct the sigma vectors of SACIS and DCIS. We restrict ourselves to real orbitals.

B. Orbital and CI gradients

1. ${}^{\rm o}g_{ai}$ and ${}^{\rm o}g^{IK}_{ai}$

The orbital gradient of CIS is

$${}^{\rm o}g_{ai} = \langle 0| \left[\hat{E}^-_{ai}, \hat{H}\right] |0\rangle \tag{S137}$$

which is computed as, using the intermediates,

$$\begin{aligned} {}^{\rm o}g_{ai} &= -(F_{ai} + F_{ia}) + [\boldsymbol{\gamma}, \mathbf{F}]_{ai} + c_{aj} G[\mathbf{c}^\top]_{ji} - G[\mathbf{c}^\top]_{ab} c_{bi} - G[\boldsymbol{\gamma}]_{ai} \\ &\quad + [\mathbf{F}, \boldsymbol{\gamma}]_{ia} - c_{bi} G[\mathbf{c}]_{ba} + G[\mathbf{c}]_{ij} c_{aj} - G[\boldsymbol{\gamma}]_{ia} \\ &= 2\big(- F_{ai} + [\boldsymbol{\gamma}, \mathbf{F}]_{ai} + c_{aj} G[\mathbf{c}^\top]_{ji} - G[\mathbf{c}^\top]_{ab} c_{bi} - G[\boldsymbol{\gamma}]_{ai}\big) \end{aligned} \tag{S138}$$

where

$$\mathbf{G}[\mathbf{c}^\top] = \mathbf{G}[\mathbf{c}]^\top \tag{S139}$$

is the 2-electron contraction (see Eq. (S19)). Using the SCF conditions, $F_{ai} = 0$ and the standard CIS amplitudes ($c_0 = 0$ and therefore $\gamma_{ai} = \gamma_{ia} = 0$),

$${}^{\rm o}g_{ai} = 2\Big(c_{aj} G[\mathbf{c}^\top]_{ji} - G[\mathbf{c}^\top]_{ab} c_{bi} - G[\boldsymbol{\gamma}]_{ai}\Big) \tag{S140}$$

Obviously, the more general Eq. (S138) has to be used for SACIS, in which the Brillouin condition is not satisfied.

Similarly to SAECIS, the transition-like quantity for the orbital gradient, ${}^{\rm o}g^{IK}_{ai} = \langle 0_I| \left[\hat{E}^-_{ai}, \hat{H}\right] |0_K\rangle$ is needed, as will be seen. This is simply obtained by using $\mathbf{c}^I$ for the bra and $\mathbf{c}^K$ the ket state. Defining the transition density matrix

$$\gamma^{IK}_{ji} = -c^I_{bj} c^K_{bi} \tag{S141}$$

$$\gamma^{IK}_{ai} = c^I_0 c^K_{ai} \tag{S142}$$

$$\gamma^{IK}_{ia} = c^K_0 c^I_{ai} \tag{S143}$$

$$\gamma^{IK}_{ab} = c^K_{aj} c^I_{bj} \tag{S144}$$

and the overlap

$$N_{IK} = \langle 0_I | 0_K \rangle = c^I_0 c^K_0 + c^I_{ai} c^K_{ai} \tag{S145}$$

it is straightforward to extend the result of Eq. (S138):

$$\begin{aligned} {}^{\rm o}g^{IK}_{ai} &= -N_{IK}(F_{ai} + F_{ia}) + [\boldsymbol{\gamma}^{IK}, \mathbf{F}]_{ai} + c^K_{aj} G[\mathbf{c}^{I,\top}]_{ji} - G[\mathbf{c}^{I,\top}]_{ab} c^K_{bi} - G[\boldsymbol{\gamma}^{IK}]_{ai} \\ &\quad + [\mathbf{F}, \boldsymbol{\gamma}^{IK}]_{ia} - c^I_{bi} G[\mathbf{c}^K]_{ba} + G[\mathbf{c}^K]_{ij} c^I_{aj} - G[\boldsymbol{\gamma}^{IK}]_{ia} \end{aligned} \tag{S146}$$

In the following, we will not explicitly write the state labels I and K, as the rest quantities depend only on a single state but do not require transition-like elements.

2. ${}^{\rm c}g_\mu$

This is simply the sigma-vector of standard CIS.

$${}^{\rm c}g_0 = 2\Big((E_{\rm HF} - E_0)c_0 + c_{ai} F_{ia}\Big) \tag{S147}$$

$${}^{\rm c}g_{ai} = 2\Big((E_{\rm HF} - E_0)c_{ai} + c_0 F_{ai} + F_{ab} c_{bi} - c_{aj} F_{ji} + G[\mathbf{c}]_{ai}\Big) \tag{S148}$$

or using the HF canonical orbitals,

$${}^{\rm c}g_0 = 0 \tag{S149}$$

$${}^{\rm c}g_{ai} = 2\Big((E_{\rm HF} - E_0)c_{ai} + (\epsilon_a - \epsilon_i)c_{ai} + G[\mathbf{c}]_{ai}\Big) \tag{S150}$$

C. Hessian contraction

The general result for the SACIS Hessian is, using the results in the main text,

$$
\begin{aligned}
{}^{\mathrm{oo}}H_{ai,bj} &= \frac{\partial^2 E_{\mathrm{ave}}}{\partial\, {}^{\mathrm{o}}\lambda_{ai} \partial\, {}^{\mathrm{o}}\lambda_{bj}}\bigg|_{\boldsymbol{\lambda}=\mathbf{0}} \\
&= \frac{1}{n}\sum_{I}^{n}\left(\frac{1}{2}\langle 0_I|\left[\hat{E}^{-}_{ai},\left[\hat{E}^{-}_{bj},\hat{H}\right]\right]|0_I\rangle + \frac{1}{2}\langle 0_I|\left[\hat{E}^{-}_{bj},\left[\hat{E}^{-}_{ai},\hat{H}\right]\right]|0_I\rangle\right)
\end{aligned} \tag{S151}
$$

$$
\begin{aligned}
{}^{\mathrm{oc}}H^{I}_{ai,\mu} &= \frac{\partial^2 E_{\mathrm{ave}}}{\partial\, {}^{\mathrm{o}}\lambda_{ai} \partial\, {}^{\mathrm{c}}\lambda^{I}_{\mu}}\bigg|_{\boldsymbol{\lambda}=\mathbf{0}} \\
&= \frac{1}{n}\left(\langle 0_I|\left[\hat{E}^{-}_{ai},\hat{H}\right]|\Phi_\mu\rangle + \langle \Phi_\mu|\left[\hat{E}^{-}_{ai},\hat{H}\right]|0_I\rangle - \sum_{K}^{n} c^{K}_{\mu}\left({}^{\mathrm{o}}g^{IK}_{ai} + {}^{\mathrm{o}}g^{KI}_{ai}\right)\right)
\end{aligned} \tag{S152}
$$

$$
\begin{aligned}
{}^{\mathrm{cc}}H^{IJ}_{\mu\nu} &= \frac{\partial^2 E_{\mathrm{ave}}}{\partial\, {}^{\mathrm{c}}\lambda^{I}_{\mu} \partial\, {}^{\mathrm{c}}\lambda^{J}_{\nu}}\bigg|_{\boldsymbol{\lambda}=\mathbf{0}} \\
&= \frac{\delta_{IJ}}{n}\Bigg(\langle\Phi_\mu|(\hat{H}-E_{0,I})|\Phi_\nu\rangle - \sum_{K}^{n} c^{K}_{\mu}\langle 0_K|(\hat{H}-E_{0,K})|\Phi_\nu\rangle - \sum_{K}^{n}\langle\Phi_\mu|(\hat{H}-E_{0,I})|0_K\rangle c^{K}_{\mu} \\
&+ \langle\Phi_\nu|(\hat{H}-E_{0,I})|\Phi_\mu\rangle - \sum_{K}^{n} c^{K}_{\mu}\langle 0_K|(\hat{H}-E_{0,K})|\Phi_\mu\rangle - \sum_{K}^{n}\langle\Phi_\nu|(\hat{H}-E_{0,I})|0_K\rangle c^{K}_{\mu}\Bigg)
\end{aligned} \tag{S153}
$$

For real orbitals,

$$
{}^{\mathrm{oo}}H_{ai,bj} = \frac{1}{n}\sum_{I}^{n} {}^{\mathrm{oo}}H^{I}_{ai,bj} \tag{S154}
$$

$$
\begin{aligned}
{}^{\mathrm{oo}}H^{I}_{ai,bj} &= \mathcal{P}(ai)\mathcal{P}(bj)(2\, {}^{\mathrm{oo}}A^{I}_{ai,bj} + {}^{\mathrm{oo}}B^{I}_{ai,bj} + {}^{\mathrm{oo}}B^{I}_{bj,ai}) \\
&= \mathcal{P}(ai)\mathcal{P}(bj)(2\, {}^{\mathrm{oo}}A^{I}_{ai,bj} + 2{}^{\mathrm{oo}}B^{I}_{ai,bj}) + (L_{ji}+L_{ij})\delta_{ab} - (L_{ab}+L_{ba})\delta_{ij}
\end{aligned} \tag{S155}
$$

$$
{}^{\mathrm{oc}}H_{ai,\nu} = -\frac{2}{n}\mathcal{P}(ai)({}^{\mathrm{oc}}A^{I}_{ai,\mu} + {}^{\mathrm{oc}}B^{I}_{ai,\mu}) - \frac{2}{n}\sum_{K}^{n} {}^{\mathrm{o}}g^{IK}_{ai} c^{K}_{\mu} \tag{S156}
$$

$$
{}^{\mathrm{cc}}H^{IJ}_{\mu\nu} = \frac{\delta_{IJ}}{n}\left(2{}^{\mathrm{cc}}A^{I}_{\mu\nu} - \sum_{K}^{n}\left({}^{\mathrm{c}}g^{K}_{\mu}c^{K}_{\nu} + {}^{\mathrm{c}}g^{K}_{\nu}c^{K}_{\mu} + 2(E_{0,K}-E_{0,I})c^{K}_{\mu}c^{K}_{\nu}\right)\right) \tag{S157}
$$

We intentionally use some trial vectors x_{ai} for the orbital rotation part and $y_\nu \in \{y_0, y_{ai}\}$ for the CI part, so that the results below can be easily generalized to both SACIS and DCIS.

1. ${}^{\mathrm{cc}}H_{\mu,\nu}y_\nu$

The contraction is the same form as the sigma-vector of standard CIS. We may define

$$
|\mathbf{y}\rangle = y_0|\Phi_0\rangle + \sum_{ai} y_{ai}|\Phi^{a}_{i}\rangle \tag{S158}
$$

then

$$
{}^{\mathrm{cc}}A_{\mu,\nu}y_\nu = \langle\Phi_\mu|(\hat{H}-E_0)|\mathbf{y}\rangle \tag{S159}
$$

This is essentially the same as ${}^{\mathrm{c}}g_\mu$ except for the pre-factor, which is 1. Therefore, we just replace (c_0, c_{ai}) in Eqs.(S147) and (S148) with (y_0, y_{ai}) (with a pre-factor of 1):

$$
({}^{\mathrm{cc}}\mathbf{Ay})_0 = (E_{\mathrm{HF}} - E_0)y_0 + y_{ai}F_{ia} \tag{S160}
$$

$$
({}^{\mathrm{cc}}\mathbf{Ay})_{ai} = (E_{\mathrm{HF}} - E_0)y_{ai} + y_0F_{ai} + y_{bi}F_{ab} - y_{aj}F_{ji} + \langle aj||ib\rangle y_{bj} \tag{S161}
$$

and for the canonical HF orbitals,

$$
({}^{\mathrm{cc}}\mathbf{Ay})_0 = (E_{\mathrm{HF}} - E_0)y_0 \tag{S162}
$$

$$
({}^{\mathrm{cc}}\mathbf{Ay})_{ai} = (E_{\mathrm{HF}} - E_0)y_{ai} + (\epsilon_a - \epsilon_i)y_{ai} + \langle aj||ib\rangle y_{bj} \tag{S163}
$$

2. ${}^{\rm oc}H_{ai,\nu}y_\nu$

Next, we consider the sigma-vectors for orbital-CI coupling. Since

$$ {}^{\rm oc}H^I_{ai,\mu}y_\mu = \frac{2}{n}\left(\langle 0_I|\left[\hat{E}^-_{ai},\hat{H}\right]|\mathbf{y}\rangle - \sum_K^n (c^K_\mu y_\mu)^{\rm o} g^{IK}_{ai}\right) \tag{S164}$$

The first term can be evaluated using the result Eq. (S64). This can be done by defining a transition-density-like matrix

$$\gamma^y_{ji} = -c_{bj}y_{bi} \tag{S165}$$
$$\gamma^y_{ai} = c_0 y_{ai} \tag{S166}$$
$$\gamma^y_{ia} = y_0 c_{ai} \tag{S167}$$
$$\gamma^y_{ab} = y_{aj}c_{bj} \tag{S168}$$

and

$$N_y = y_0c_0 + y_{bj}c_{bj} \tag{S169}$$

and by simply replacing N_{IK} and γ^{IK}_{pq} by N_y and γ^y_{pq}. Here, the label I is omitted from $\mathbf{c}$ for simplicity.

For DCIS, we also need the explicit contribution of ${}^{\rm oc}A_{ai,\nu}y_\nu$ (without the $\mathbf{B}$ term). It is relatively easy to show

$$\begin{aligned}({}^{\rm oc}\mathbf{Ay})_{ai} &= \langle 0|\hat{E}_{ia}(\hat{H}-E_0)|\mathbf{y}\rangle \\ &= (E_{\rm HF}-E_0)\gamma^y_{ai} + N_yF_{ai} + y_{ai}[c_{bj}F_{bj}] + F_{ap}\gamma^y_{pi} - \gamma^y_{ap}F_{pi} + G[\mathbf{c}^\top]_{ab}y_{bi} - y_{aj}G[\mathbf{c}^\top]_{ji} + G[\boldsymbol{\gamma}^y]_{ai}\end{aligned} \tag{S170}$$

Here, $E_{\rm HF} = \langle\Phi_0|\hat{H}|\Phi_0\rangle$ is the HF-energy-like value. In the canonical orbital basis, $c_0 = 0$ and $\gamma^y_{ai} = 0$ but $y_0 \neq 0$ and therefore $\gamma^y_{ia} \neq 0$ in general;

$$({}^{\rm oc}\mathbf{Ay})_{ai} = G[\mathbf{c}^\top]_{ab}y_{bi} - y_{aj}G[\mathbf{c}^\top]_{ji} + G[\boldsymbol{\gamma}^y]_{ai} \tag{S171}$$

3. ${}^{\rm co}H_{\mu,bj}x_{bj}$

For this term, we cannot use the trick of $|\mathbf{y}\rangle$ unlike above. Nevertheless, its derivation is straightforward. Let us begin with the following equation:

$$ {}^{\rm co}H^I_{\mu,bj}x_{bj} = \frac{2}{n}\left(\langle\Phi_\mu|\left[\hat{E}^-_{bj}x_{bj},\hat{H}\right]|0_I\rangle - \sum_K^n c^K_\mu ({}^{\rm o}g^{IK}_{ai}x_{bj})\right) \tag{S172}$$

We introduce another intermediate N_x and $\boldsymbol{\gamma}^x$, similarly to N_y and $\boldsymbol{\gamma}^y$:

$$N_x = c_{bj}x_{bj} \tag{S173}$$
$$\gamma^x_{jk} = -c_{cj}x_{ck} \tag{S174}$$
$$\gamma^x_{ba} = x_{bj}c_{aj} \tag{S175}$$
$$\tag{S176}$$

where $\gamma^x_{ai} = \gamma^x_{ia} = 0$. The first term of Eq.(S172) then becomes

$$\langle\Phi_0|\left[\hat{E}^-_{bj}x_{bj},\hat{H}\right]|0_I\rangle = -c_0(F_{ia}+F_{ai})x_{ai} + c_{ai}(F_{ij}x_{aj} - x_{bi}F_{ba}) - c_{ai}G[\mathbf{x}]_{ia} - c_{ai}G[\mathbf{x}]_{ai} \tag{S177}$$

$$\begin{aligned}\langle\Phi^a_i|\left[\hat{E}^-_{bj}x_{bj},\hat{H}\right]|0_I\rangle &= c_0(x_{aj}F_{ji} - F_{ab}x_{bi} - G[\mathbf{x}]_{ai} - G[\mathbf{x}]_{ia}) \\ &\quad - c_{ai}(F_{jb}+F_{bj})x_{bj} + c_{aj}(F_{jb}x_{bi} + x_{bj}F_{bi}) + (x_{aj}F_{jb} + F_{aj}x_{bj})c_{bi} \\ &\quad + c_{aj}(G[\mathbf{x}]_{ji} + G[\mathbf{x}]_{ij}) - (G[\mathbf{x}]_{ab} + G[\mathbf{x}]_{ba})c_{bi} + x_{aj}G[\mathbf{c}]_{ji} - G[\mathbf{c}]_{ab}x_{bi} - G[\boldsymbol{\gamma}^x]_{ia}\end{aligned} \tag{S178}$$

The sigma-vectors for DCIS, $({}^{\rm co}\mathbf{Ax})_\mu = \langle\Phi_\mu|(\hat{H}-E_0)\hat{E}_{ai}|0\rangle x_{ai}$, can be readily obtained as

$$({}^{\rm co}\mathbf{Ax})_0 = c_0[F_{ia}x_{ai}] + G[\mathbf{c}]_{jb}x_{bj} \tag{S179}$$

$$\begin{aligned}({}^{\rm co}\mathbf{Ax})_{ai} &= c_0\big((E_{\rm HF}-E_0)x_{ai} + F_{ab}x_{bi} - x_{aj}F_{ji} + G[\mathbf{x}]_{ai}\big) + c_{ai}(F_{jb}x_{bj}) - c_{aj}F_{jb}x_{bi} - x_{aj}F_{jb}c_{bi} + x_{ai}(c_{bj}F_{jb}) \\ &\quad - c_{aj}G[\mathbf{x}]_{ji} + G[\mathbf{x}]_{ab}c_{bi} - x_{aj}G[\mathbf{c}]_{ji} + G[\mathbf{c}]_{ab}x_{bi}\end{aligned} \tag{S180}$$

and enforcing $c_0 = F_{jb} = F_{bj} = 0$ for the canonical HF orbitals,

$$({}^{\rm co}\mathbf{Ax})_0 = G[\mathbf{c}]_{jb}x_{bj} \tag{S181}$$
$$({}^{\rm co}\mathbf{Ax})_{ai} = G[\mathbf{x}]_{ab}c_{bi} - c_{aj}G[\mathbf{x}]_{ji} + G[\mathbf{c}]_{ab}x_{bi} - x_{aj}G[\mathbf{c}]_{ji} \tag{S182}$$

4. ${}^{\mathrm{oo}}H_{ai,bj}x_{bj}$

Finally, we will provide detailed equation for the sigma-vector ${}^{\mathrm{oo}}H_{ai,bj}x_{bj}$. It is useful to define an additional intermediate:

$$\mathbf{u} = [\mathbf{x}, \boldsymbol{\gamma}] \tag{S183}$$

In other words,

$$u_{jk} = -\gamma_{jb}x_{bk} \quad \text{(0 for canonical HF)} \tag{S184}$$
$$u_{bj} = x_{bk}\gamma_{kj} - \gamma_{bc}x_{cj} \tag{S185}$$
$$u_{jb} = 0 \tag{S186}$$
$$u_{cb} = x_{cj}\gamma_{jb} \quad \text{(0 for canonical HF)} \tag{S187}$$

Let us first provide the contraction for DCIS, i.e., ${}^{\mathrm{oo}}A_{ai,bj}x_{bj}$. Our result is

$$\begin{aligned}
({}^{\mathrm{oo}}\mathbf{Ax})_{ai} &= \langle 0|\hat{E}_{ia}(\hat{H} - E_0)\hat{E}_{bj}|0\rangle x_{bj} \\
&= (E_{\mathrm{HF}} - E_0)\Big(c_{ai}N_x + u_{ai}\Big) \\
&+ F_{ai}(\gamma_{jb}x_{bj}) + (F_{ab}c_{bi} - c_{aj}F_{ji})N_x \\
&+ (F_{ab} - F_{ap}\gamma_{pb} - \gamma_{ap}F_{pb})x_{bi} - (F_{ac}\gamma^x_{cb} + \gamma^x_{ac}F_{cb})c_{bi} - x_{ak}(F_{ki} + F_{kp}\gamma_{pi} + \gamma_{kp}F_{pi}) - c_{aj}(\gamma^x_{jk}F_{ki} + F_{jk}\gamma^x_{ki}) \\
&+ \gamma_{ai}[F_{jb}x_{bj}] + c_{ai}(\gamma^x_{qp}F_{pq}) \\
&+ G[\mathbf{u}]_{ai} + G[\mathbf{x}]_{ai} + c_{ai}(c_{bj}G[\mathbf{x}]_{bj}) - c_{aj}G[\mathbf{c}^\top]_{jb}x_{bi} - x_{aj}G[\mathbf{c}^\top]_{jb}c_{bi} + G[\mathbf{c}]_{ai}N_x \\
&+ G[\boldsymbol{\gamma}]_{ab}x_{bi} - x_{aj}G[\boldsymbol{\gamma}]_{ji} + G[\mathbf{x}]_{ap}\gamma_{pi} - \gamma_{ap}G[\mathbf{x}]_{pi} + G[\mathbf{c}]_{aj}\gamma^x_{ji} - \gamma^x_{ab}G[\mathbf{c}]_{bi} + G[\boldsymbol{\gamma}^x]_{ab}c_{bi} - c_{aj}G[\boldsymbol{\gamma}^x]_{ji}
\end{aligned} \tag{S188}$$

When the HF canonical orbitals are used,

$$\begin{aligned}
({}^{\mathrm{oo}}\mathbf{Ax})_{ai} &= (E_{\mathrm{HF}} - E_0)\Big(c_{ai}N_x + u_{ai}\Big) \\
&+ (\epsilon_a - \epsilon_i)\,(x_{ai} + N_x c_{ai}) + c_{ai}[\gamma^x_{pp}\epsilon_p] \\
&- (\epsilon_a + \epsilon_b)(\gamma_{ab}x_{bi} + \gamma^x_{ab}c_{bi}) - (\epsilon_i + \epsilon_j)(c_{aj}\gamma^x_{ji} + x_{aj}\gamma_{ji}) \\
&+ G[\mathbf{u}]_{ai} + G[\mathbf{x}]_{ai} + c_{ai}(c_{bj}G[\mathbf{x}]_{bj}) - c_{aj}G[\mathbf{c}^\top]_{jb}x_{bi} - x_{aj}G[\mathbf{c}^\top]_{jb}c_{bi} + G[\mathbf{c}]_{ai}N_x \\
&+ G[\boldsymbol{\gamma}]_{ab}x_{bi} - x_{aj}G[\boldsymbol{\gamma}]_{ji} + G[\mathbf{x}]_{aj}\gamma_{ji} - \gamma_{ab}G[\mathbf{x}]_{bi} + G[\mathbf{c}]_{aj}\gamma^x_{ji} - \gamma^x_{ab}G[\mathbf{c}]_{bi} + G[\boldsymbol{\gamma}^x]_{ab}c_{bi} - c_{aj}G[\boldsymbol{\gamma}^x]_{ji}
\end{aligned} \tag{S189}$$

Now, we need other terms for the Hessian contraction; for example,

$$\begin{aligned}
{}^{\mathrm{oo}}B_{ai,bj}x_{bj} &= \gamma_{ia}(F_{jb}x_{bj}) + (\gamma_{jb}x_{bj})F_{ia} + u_{ij}F_{ja} - F_{ib}u_{ba} + N_xG[\mathbf{c}]_{ia} + \gamma_{ib}G[\mathbf{x}]_{ba} - G[\mathbf{x}]_{ij}\gamma_{ja} + c_{ai}(c_{bj}G[\mathbf{x}]_{jb}) \\
&+ \gamma_{ij}G[\mathbf{x}]_{ja} - G[\mathbf{x}]_{ib}\gamma_{ba} + \gamma^x_{ik}G[\mathbf{c}]_{ka} - G[\mathbf{c}]_{ic}\gamma^x_{ca} + G[\mathbf{x}]_{ia} + G[\mathbf{u}]_{ia}
\end{aligned} \tag{S190}$$

Summarizing all terms, we find

$$\begin{aligned}
{}^{\mathrm{oo}}H_{ai,bj}x_{bj} &= 2([\mathbf{F},\mathbf{u}]_{ai} - [\mathbf{F},\mathbf{u}]_{ia}) + 2\,[\mathbf{F},\mathbf{x}]_{ai} + [[\mathbf{F},\boldsymbol{\gamma}]\,,\mathbf{x}]_{ai} + 2G[\mathbf{u}]_{ai} + 2G[\mathbf{u}]_{ia} + 2G[\mathbf{x}]_{ai} + 2G[\mathbf{x}]_{ia} - (\mathbf{xG}[\mathbf{c}^\top]\mathbf{c})_{ai} \\
&- (\mathbf{cG}[\mathbf{c}^\top]\mathbf{x})_{ai} + 2\,[\mathbf{G}[\mathbf{x}],\boldsymbol{\gamma}]_{ai} - 2\,[\mathbf{G}[\mathbf{x}],\boldsymbol{\gamma}]_{ia} + 2[\mathbf{G}[\boldsymbol{\gamma}],\mathbf{x}]_{ai} + 2\,[\mathbf{G}[\boldsymbol{\gamma}^x],\mathbf{c}]_{ai} + 2\,[\boldsymbol{\gamma}^x,\mathbf{G}[\mathbf{c}]]_{ia} + [\mathbf{G}[\mathbf{c}],\boldsymbol{\gamma}^x]_{ai}
\end{aligned} \tag{S191}$$
